# Predicting acute kidney injury at hospital re-entry using high-dimensional electronic health record data

**DOI:** 10.1371/journal.pone.0204920

**Published:** 2018-11-20

**Authors:** Samuel J. Weisenthal, Caroline Quill, Samir Farooq, Henry Kautz, Martin S. Zand

**Affiliations:** 1 Rochester Center for Health Informatics, University of Rochester Medical Center, Rochester, NY, United States of America; 2 Clinical Translational Science Institute, University of Rochester Medical Center, Rochester, NY, United States of America; 3 Department of Medicine, Division of Nephrology, University of Rochester Medical Center, Rochester, NY, United States of America; 4 Department of Medicine, Division of Pulmonary and Critical Care Medicine, University of Rochester Medical Center, Rochester, NY, United States of America; 5 Department of Computer Science, University of Rochester, Rochester, NY, United States of America; 6 Goergen Institute for Data Science, University of Rochester, Rochester, NY, United States of America; University of Sao Paulo Medical School, BRAZIL

## Abstract

Acute Kidney Injury (AKI), a sudden decline in kidney function, is associated with increased mortality, morbidity, length of stay, and hospital cost. Since AKI is sometimes preventable, there is great interest in prediction. Most existing studies consider all patients and therefore restrict to features available in the first hours of hospitalization. Here, the focus is instead on rehospitalized patients, a cohort in which rich longitudinal features from prior hospitalizations can be analyzed. Our objective is to provide a risk score directly at hospital re-entry. Gradient boosting, penalized logistic regression (with and without stability selection), and a recurrent neural network are trained on two years of adult inpatient EHR data (3,387 attributes for 34,505 patients who generated 90,013 training samples with 5,618 cases and 84,395 controls). Predictions are internally evaluated with 50 iterations of 5-fold grouped cross-validation with special emphasis on calibration, an analysis of which is performed at the patient as well as hospitalization level. Error is assessed with respect to diagnosis, race, age, gender, AKI identification method, and hospital utilization. In an additional experiment, the regularization penalty is severely increased to induce parsimony and interpretability. Predictors identified for rehospitalized patients are also reported with a special analysis of medications that might be modifiable risk factors. Insights from this study might be used to construct a predictive tool for AKI in rehospitalized patients. An accurate estimate of AKI risk at hospital entry might serve as a prior for an admitting provider or another predictive algorithm.

## Introduction

Acute kidney injury (AKI) is a sudden decline in kidney function over days, which may be temporary or permanent [1, 2]. AKI is common in hospitalized patients, with an estimated incidence of 13% and, importantly, is associated with greatly increased morbidity (e.g., long-term dialysis), mortality, length of stay, and hospital cost [3]. Diagnosis of AKI is challenging, as patients are generally asymptomatic and commonly used biomarkers change over a period of days following injury [4]. Causes of AKI are generally grouped into decreased renal blood flow (e.g., hypotension due to sepsis or heart failure), direct renal toxicity (e.g., due to medications, radiocontrast dye, or bacterial toxins), and urinary outflow obstruction (e.g., bladder outlet obstruction or kidney stones).

Defining AKI for research purposes, or to assess clinical outcomes, is also challenging. A variety of definitions exist, primarily based on changes in the concentration of serum creatinine (sCr). Creatinine is a protein made by muscle and excreted by the kidneys via glomerular filtration. Serum Cr is inversely proportional to the glomerular filtration rate (GFR), a true indicator of renal function that is not easily measured. Doubling of sCr at steady state reflects a 50% decrease in renal function. Consensus definitions for AKI rely heavily on changes in sCr over time, and include the 2004 RIFLE criteria [1] (modified in 2007 by the Acute Kidney Injury Network (AKIN) [5]) and the 2012 Kidney Disease: Improving Global Outcomes (KDIGO) [6, 7] definitions. The KDIGO AKI definition, which we use here, combines the RIFLE “Risk” definition with the AKIN criterion for absolute increase in sCr.

Developing a broadly applicable and accurate risk index for AKI in rehospitalized patients could have a major impact on hospital care, particularly if it were practical enough to allow preventive intervention or more intense monitoring from the time of hospital admission [8]. With early risk identification, a variety of preventive strategies can be implemented [9]. For example, AKI from radiocontrast dye, chemotherapy, or aminoglycoside antibiotics can be prevented by altering treatment, administration of fluids, alternate imaging modalities or close monitoring [10–13]. Given that such interventions can mitigate severity, AKI prediction is an area of active research, with recent emphasis on Electronic Health Record (EHR) data [8, 14–16]. Existing studies generally focus on AKI in the context of cardiac procedures [17–20], critical illness [21–26], the elderly [27], transplants of the liver [28] and lung [29], and extensive muscle injury (rhabdomyolysis) [30, 31]. Recently, we see predictive systems [15, 16, 27] that exploit numerous features from the EHR rather than a small number of manually picked variables. In existing studies, predictions of AKI risk are made for *all* hospitalized patients, many of whom do not have previous hospitalizations. They are hence restricted to features from the current hospitalization, even when a patient has more extensive information in the EHR.

To our knowledge, there are no published studies focused explicitly and exclusively on a large cohort of *rehospitalized* patients. Focus on this group allows analysis of longitudinal information from prior hospitalizations (e.g., the number of previous episodes of AKI, the number of abnormal urea nitrogen (UN) readings, or the number of loop diuretics administered). Although the subset of rehospitalized patients is a specific cohort, such an analysis is general as it pertains to *all* rehospitalized patients. Since rehospitalized patients have not been studied explicitly in the literature, all available features from all available time points were analyzed.

In this framework, prior hospitalizations might be considered surrogate “renal stress tests,” reflecting renal resiliency to injurious events. Conversely, prior hospitalizations might be renal stressors, diminishing renal reserve. Most previous studies on AKI posit data models, although some more recent work [16, 27] explores predictive algorithms, distinct from data models [32], as done here. Penalized regression and ensemble methods were employed to mitigate overfitting. In particular, a decision tree ensemble classifier constructed with gradient boosting (GBC), which is highly robust to outliers and well suited to high-dimensional, noisy data [33], was explored along with a recurrent neural network [34] (LSTM) for time series analysis, and penalized logistic regression (LR1) for high-dimensional data where it is believed that only a few features are relevant [35]. In an additional experiment with the latter, the penalty was increased severely to induce parsimony and interpretability. New AKI predictors specific to rehospitalized patients were identified; a special analysis of medication-related predictors is presented as they may be of interest as potentially modifiable risk factors.

## Materials and methods

### Dataset

The research protocol was approved by the University of Rochester Research Subjects Review Board (RSRB00056930). Research data were coded such that patients could not be directly identified in compliance with the Department of Health and Human Services Regulations for the Protection of Human Subjects (45 CFR 46.101(b)(4)). This dataset is a comprehensive 2-year window into the EHR for informatics and population-health studies. For this work we excluded all hospitalizations with age at admission < 18 years, all hospitalizations following a prior hospitalization in which the ICD-9 code for end stage renal disease (ESRD; 585.9) was assigned. Hospitalizations following a transplant for ESRD were included. Patients who had undergone dialysis were included, as dialysis is sometimes performed in the setting of transient AKI, and therefore presence of dialysis does not indicate permanent renal dysfunction. Multiple hospitalizations were available for roughly 32% of patients.

The dataset consisted of tables containing administrative, laboratory, and medication data that was queried respectively from separate billing (Flowcast, IDX Systems), eRecord (Epic), and pharmacy databases which could be joined on admit id, which were linked during de-identification. The administrative dataset included International Classification of Diseases, 9^th^ Revision (ICD-9) diagnosis and procedure codes, Current Procedural Terminology 4th Edition (CPT-4) procedure codes, Diagnosis-Related Groupings (DRG) codes, bed locations during hospitalization, discharge disposition, discharge and admission days, insurance (primary, secondary, and other), marital status, gender, age, race, and total length of stay. The laboratory dataset included direct bilirubin, point-of-care creatinine, bicarbonate, chloride, calcium, anion gap, phosphate, glomerular filtration rate, sCr, urea nitrogen (UN), albumin, total protein, aspartate and alanine transaminase, hemoglobin, glucose, and glycated hemoglobin. The pharmacy dataset included, for each medication, description, pharmacologic class and subclass, and therapeutic class. Table 1 contains abbreviations.

**Table 1:** Abbreviations and Notation.

Abbreviation	Description
AKI	Acute kidney injury
ALR1	Anscombe LR1
AUC	Area under the curve
CKD	Chronic kidney disease
CLR	Clinical LR
CV	Cross validation
Dx	Diagnosis
EHR	Electronic health record
ESRD	End-stage renal disease
GBC	Gradient boosting classifier
GFR	Glomerular filtration rate
RGBC	Recent GBC
RHPLR1	Randomized HPLR1
RLR1	Randomized LR1
*H*_*C*_	Current hospitalization
*H*_*P*_	Prior hospitalizations
HP	Hyperparameter
HPLR1	Highly penalized LR1
LR1	Logistic regression with *l*1-norm penalty
LSTM	Long short-term memory
MGBC	Medication GBC
MLR1	Medication LR1
PPV	Positive predictive value
*P*_*P*_	Predicted probability
$\bar{P_{P}}$	Mean *P*_*P*_
*P*_*O*_	Observed probability
$\bar{P_{O}}$	Mean *P*_*O*_
PR	Precision recall
Px	Procedure
ROC	Receiver operating characteristic
sCr	Serum creatinine
STD	Standard deviation
UN	Urea nitrogen

### Definitions

*Hospitalization*: hospitalization was defined as an admission (inpatient) or administrative status “under observation” (e.g., in the emergency department, but not admitted to inpatient care). *AKI*: AKI was defined as the presence of either an administrative diagnosis code or sCr delta. Administrative ICD9 codes included 584.5 (AKF with lesion of tubular necrosis), 584.6 (AKF with lesion of renal cortical necrosis), 584.7 (AKF with lesion of renal medullary (papillary) necrosis), 584.8 (AKF with other specified pathological lesion in kidney), or 584.9 (AKF, unspecified). As diagnosis codes are believed to be specific but not sensitive for AKI [36], they were supplemented with sCr for patients with available laboratory values. Using KDIGO guidelines [6], diagnosis was made with a 1.5-fold or greater increase in sCr from baseline within 7 days or 0.3 mg/dL or greater increase in sCr within 48 hours. Baseline sCr for an individual hospital stay was defined as the first documented inpatient sCr, as recommended by [7], and then as a sliding baseline. All such diagnoses were made within a single hospital stay (e.g., a case in which the rise in sCr occurred over two rapidly successive hospital stays was ignored). It is possible that some patients who did have AKI were neither assigned a code nor had their sCr measured, and would thus be invisible with respect to AKI diagnosis.

### Preprocessing

Medication descriptions were stripped of dosage information and treated as categorical variables. Abnormal lab flags were constructed by combining EHR-generated flags (an automated indication of, e.g., hyperkalemia) with test name. For features such as diagnoses and procedures, each admission contained a list. The hospitalization with the highest number, *D*, of diagnoses was identified. Given any other hospitalization with a list of *D*′ diagnoses, *D* − *D*′ “non-diagnoses,” the number of diagnoses not assigned relative to its peers, were added. This was done to enhance predictive performance, as missingness patterns were useful for the related task of phenotyping [37]. Top-level binary representation [38] was used for the hierarchical ICD-9, CPT-4, and DRG codes and “code precision” is defined as the level at which the tree was accessed. For example, for chronic kidney disease (CKD), precision 3 produces a single feature, 581, that contains any occurrence of 585.1-6 (CKD, Stages 1-6) or 585.9 (CKD, unspecified), essentially grouping these codes. Alternatively, precision 4 ungroups the subcodes, producing 7 different features, one for each administrative classification of CKD. An exploratory grid search for precision of ICD-9, CPT-4, and DRG codes was performed and discrimination found to be relatively insensitive, so code precision was fixed at 3, a level at which different subgroups of AKI and CKD were aggregated.

All extremely sparse features (with fewer than 100 non-zero or non-missing elements) were removed. Hence, for continuous values, features that were unobserved frequently or frequently zero (continuous lab and demographic values in this dataset should generally be nonzero) were removed; categorical variables that were rarely observed were removed. Besides reducing training time, removing rarely-present features, which can be difficult to gather, improves clinical applicability. This step did not need to be incorporated into the pipeline as it was a form of response-independent dimensionality reduction.

### Feature extraction

**Fig 1:**
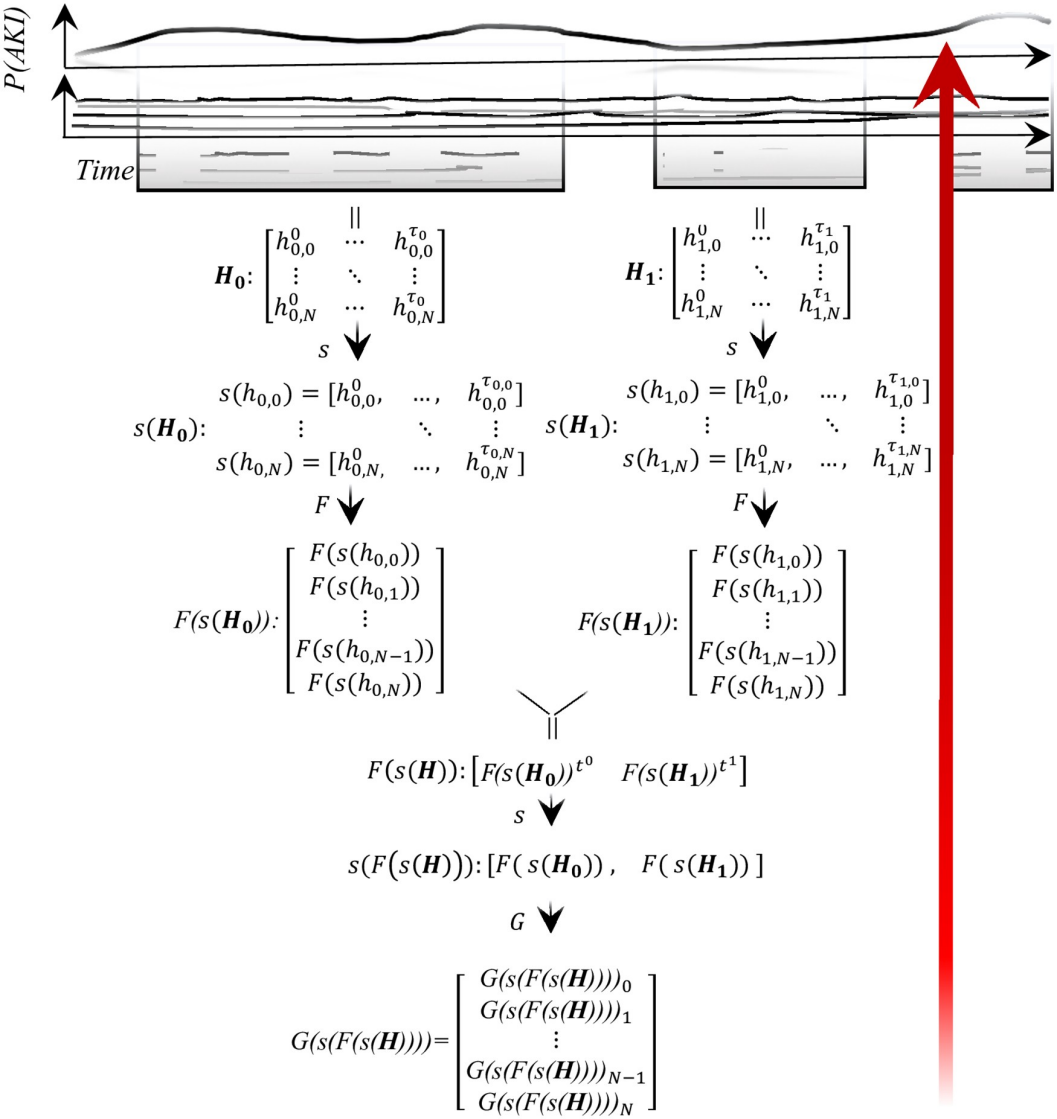
Feature extraction pipeline for estimating *P*(*AKI*) during rehospitalization. We sought to estimate the probability of AKI during rehospitalization given all of a patient’s previous hospitalizations, $\text{P}\left({\text{AKI}}_{H_{C}}|H_{P}\right)$), shown by the red arrow. An example of three hospitalizations (*H*_1_, *H*_2_, *H*_3_) is shown. Here *H*_1_ and *H*_2_ are used to estimate *P*(*AKI*) during *H*_3_. The EHR captures raw data (shown in boxes closest to the time series tracings) of which our dataset contains *N* + 1 features. At each level, data is aggregated via domain-expertise-informed functions *F* and *G*. The pipeline produces a single, fixed-length representation of all previous hospitalizations to serve as input to a learning algorithm. Measurements from each hospitalization, and series of hospitalizations, are treated as sequences, denoted with operator *s*.

Over time, all patients have some continuous risk, *P*(*AKI*). Using data from all previous hospitalizations, *H*_*P*_, we hope to estimate the probability of AKI during the current hospitalization, *H*_*C*_, at the time of hospital re-entry, $\text{P}\left({\text{AKI}}_{H_{C}}|H_{P}\right)$). An example case illustrating the feature extraction procedure used throughout this study is diagrammed in Fig 1. It was designed to compress longitudinal, irregular and misaligned observations into a fixed-length representation. Summary statistics are used for repeated measures since they are interpretable and shown by [39] to be effective for some risk prediction problems. Note that a patient with *n* hospitalizations generates *n* − 1 training samples.

EHR data is an irregularly sampled (e.g., sCr is *not* measured at an hourly frequency), misaligned (e.g., sCr and hemoglobin are *not* consistently simultaneously sampled) window into a patient’s renal health. The *j*^*th*^ hospitalization can be conceptualized as a matrix *H*_*j*_ where each row is one of *N* + 1 features and each column corresponds to some time step ≤ *τ*_*j*_ + 1, the end time of hospitalization *j*. Since observations are irregularly sampled and misaligned, it is convenient to transform the time-indexed hospitalization matrix into a collection of *N* + 1 sequences *s*(**H**), where *s* is a function that converts a time series to a sequence. The sequence *s*(*h*_*i*,*j*_) corresponds to feature *j* of hospitalization *i* and has its own number of entries, *τ*_*i*,*j*_+ 1, corresponding to the number of times feature *i* was recorded during hospitalization *j*. Such sequences are useful because they can be summarized or transformed via some function *F* without *explicit* imputation, albeit with information loss.

Summary functions can encode relevant characteristics of the generative process; e.g., a sum provides a sense of the number of tests along with some information about the results of the tests. A higher number of sCr tests may reflect a heightened concern for, or closer monitoring of, AKI and its metabolic consequences. *F* takes as input a sequence and outputs the minimum, maximum, mean, variance, and sum for continuous variables and sum for categorical variables. *F*(*s*(***H***_*i*_)) is now a *M*x1 fixed-length representation of the *N* + 1 sequences in the *i*^*th*^ hospitalization, where we have increased the dimension *M* > (*N* + 1) by concatenating any vector outputs of F.

Let **H** refer to all hospitalizations before the rehospitalization for which *P*(*AKI*) is to be estimated (e.g., the third in Fig 1). As with the sequences of laboratory measurements, *F*(*s*(***H***)) is represented as a matrix whose entries are indexed by time of admission. However, although the observations are now aligned, they are still irregularly sampled. *F*(*s*(***H***)) can then again be converted to a sequence *s*(*F*(*s*(***H***))). Finally, the sequence of hospitalizations *s*(*F*(*s*(***H***))) can be summarized, or aggregated, using *G* to yield *G*(*s*(*F*(*s*(***H***)))), a *P*-dimensional vector, where any vector outputs of *G* have been concatenated as with the hospitalization representations, so *P* > *M*. The full set of hospitalization-level aggregation functions for G is: *Administrative*: Max (age), First (race), Last (marital status, gender, insurance), Sum (DRG, discharge disposition, length of stay, locations visited, diagnoses, CPT4/ICD9 procedure); *Medications*: Sum (administration of medication by description, class, and subclass); *Labs*: Minimum, Mean, Maximum, Sum, and Variance (labs and abnormal lab flags). By aggregating over hospitalizations with *G*, most (not those that were “first” or “last”) categorical variables are rendered continuous, so standard rather than minimum-maximum scaling is used where necessary for regression.

There are benefits and drawbacks of this aggregation-based approach for time series data. Benefits include easy determination of features since *F* and *G* (chosen by the analyst) are known. The *sum* of sCr from prior hospitalizations is easy to understand; a more complicated function learned from the data in a time-dependent algorithm might not be so. A mirroring limitation is that *F* and *G*, invented by humans, are probably not optimal for the task at hand (e.g., the optimal hospital aggregator is probably a function with some weight decay over time, allowing distant events to be “forgotten”). Another drawback is loss of information on time between events (e.g., very frequent testing might be informative) or recency of events (e.g., a very distant nephrotoxic medication might be less important than a very recent one). We therefore implemented a recurrent neural network as well, but this work would likely benefit from further exploration of clinical time series methods, an active area of research [37, 40–42].

### Training

The algorithms used have hyperparameters (HP) (e.g., the number of estimators in GBC or scale of the Laplace distribution in LR1) that must be set in addition to the parameters. For learning algorithms with HP, “nested” cross-validation (CV) is recommended [43, 44] to provide an un-optimistic performance estimate. Pure nested CV requires that both choosing the HP search space and conducting the HP search be executed independently and identically within every fold. This is computationally expensive because it allows for high complexity HP (e.g., GBC with a large number of estimators or LR1 with a very small penalty) that lead to overfitting and slow training. HP were therefore fixed at values found in preliminary experiments (manually or with grid or random search [45]) to not overfit the data (as determined by a validation set distinct from the test set) and to produce reasonable features per domain expertise. It was also confirmed that performance on the test set of the fold used to determine HP did not differ substantially from performance on the test sets of the other four folds.

In greater detail, our HP selection method was as follows: create splits for 5-fold CV. Hence, we have folds 1-5, which consist of Train1, Train2, …, Train5, and Test1, Test2, …, Test5. To set HP, take Train1 and split it into a (sub)train set Train1Train and validation set Train1Val. Fit different *HP* on Train1Train and see which HP makes performance (per) for system (sys) per(sys_*HP*_(Train1Train)) ≈ per(sys_*HP*_(Train1Val)). Note that we do not try a large number of different HP and select the one with best performance on the validation set; we select the HP for which training and validation errors are most similar. Also, we examine importances/coefficients from sys_*HP*_(Train1Train) to ensure that they are reasonably related to renal function. If not, increase regularization. With this process, choose *HP*, which were found by analyzing Train1, so call them *HPTrain*1. Fix *HPTrain*1. Evaluate sys_*HPTrain*1_(Train1) on Test1, where Train1Train+Train1Val = Train1. This is a pure estimate of generalization performance. Now, keeping *HPTrain*1 fixed, evaluate sys_*HPTrain*1_(Train2) on Test2, evaluate sys_*HPTrain*1_(Train3) on Test3, and so on. Note that there is necessarily overlap between Train1 and Test2, …, Test5. Hence, there is potential leakage from *HPTrain*1 into the performance estimates of Test2, …, Test5 (but again not into Test1). During training, we therefore checked that performance on Test1 was roughly similar to performance on Test2, …, Test5.

We name this process “pseudo”-nested CV because HP selection was not performed independently in each fold as is required for pure nested CV. In pure nested CV, we would have specified a search region for HP and allowed HP to be selected in every fold, selecting *HPTrain*1 to be tested on Test1, *HPTrain*2 to be tested on Test2, and so on. Knowing that choosing our HP manually using the data put us in danger of overfitting, we purposely tried to choose *HPTrain*1 that would yield systems with lower capacity. Also, note that manual choice of HP precludes comparison of algorithms because HP choice is a confounder; our comparison is therefore over trained systems, not training algorithms.

Fixed HP for GBC included maximum depth = 2, minimum samples per split = 150, and minimum samples per leaf = 100 and for LR1 C = 2 x 10^−3^. To produce a parsimonious, highly penalized LR1 (HPLR1), C was decreased to 2 x 10^−4^ (aiming for ≈ 12 features). For LR1, classes were weighted according to prevalence. Between GBC and LR1, choice of learning algorithm was also an HP, but was wrapped into the inner folds of the nested CV as a grid search. In preliminary experiments, LR1, Ridge [46], random forest [47], multilayer perceptron [48], and GBC were explored manually. Ultimately, LR1 and GBC were chosen as candidates for the search since LR1 was close enough to ridge (the problem was expected to be sparse) and GBC close enough to random forest. As recommended in [49], log loss, rather than a binary metric, was optimized in the searches.

Although a search was performed over learning algorithms, there was no intention of comparing them outright (there are many confounders, e.g., HP choice). Rather, they were intended for use in concert since both have benefits and drawbacks. A major difference is that LR1 is linear in its parameters and therefore quite interpretable while GBC is nonlinear and sometimes gives better off-the-shelf predictions (LR1 could be enhanced with basis functions to rival, but this was not done here). Besides manual setting of HP, all other steps were performed in a pipeline within each fold. Pipelines were constructed to successively impute (using the most frequent value), scale (using standard scaling; only for LR1), fit, and calibrate (using Platt’s scaling [50]). Training data were split such that, in each fold, 75% of the observations were used to fit and select classifiers, and the remaining 25% were held out and used to calibrate the estimator with the lowest log loss. For HPLR1, there was no search over GBC.

In an additional experiment, we implemented a variance stabilizing Anscombe transform for LR1 (ALR1) for the count and categorical variables. GBC seemed to be unaffected by this transform because it is a tree-based system invariant to monotone transformations of the input. Since *l*1-norm penalty is known to select one and discard *x* − 1 of *x* highly correlated features, for the purposes of reporting features, Both LR1 and HPLR1 were rerun with stability selection [51] (these were named RLR1 and RHPLR1, respectively), which is less likely to discard the remaining *x* − 1 features. In this case, the penalty weight, *C*_2_, on the final classifier was roughly nonexistent (vanilla logistic regression) because the feature selection step with penalty weight *C*_1_ regularized. RLR1 randomized selection had *C*_1_ = 0.5 and *C*_2_ = 1; RHPLR1 had *C*_1_ = 0.2 and *C*_2_ = 1. For both RLR1 and RHPLR1, the stability selection sampling fraction was 0.75 with 50 resamples.

To explore alternative strategies for repeated measures, the first-described experiment was redone exactly, but repeated samples were weighted such that each patient received equal total representation (e.g., a patient with 3 samples was weighted by 1/3; a patient with 2 by 1/2), producing weighted GBC (WGBC), weighted LR1 (WLR1), and weighted HPLR1 (WHPLR1); alternatively, one sample per patient was randomly selected to produce independent data, producing sampled GBC (SGBC), sampled LR1 (SLR1), and sampled HPLR1 (SHPLR1). Also, for repeated measures, we implemented a recurrent neural network with long short-term memory (LSTM) cells [34] that processed the two most recent hospitalizations in sequence. This recurrent system obviated the need for the hospital aggregator *G*. We set the number of hidden layers and units *a priori* and searched over levels of dropout. Thus in contrast to GBC and LR, we did not set HP for LSTM by using features in one fold of cross validation, and therefore the LSTM was trained with pure nested cross validation. For insertion of LSTM into a pipeline, the Scikit-learn scaler and imputer were decorated to process tensors.

To explore the effect of previous hospitalizations, the first-described experiment was redone exactly, but using only the most recent hospitalization as input (results are reported for “recent” GBC: RGBC). The original decision to include data from all previous hospitalizations was based on the premise that it is better to provide more rather than less information to a learning algorithm (although this requires an extra step of aggregation over hospitalizations). As medications are potentially modifiable risk factors, GBC and LR1 were also refit exactly as in the first-described experiment, but with only medications as features (MGBC and MLR1). We also implemented a system, CGBC, with only a handful of clinically known risk factors from [52]: age, underlying renal insufficiency (prior AKI or CKD), diabetes, and heart failure. Logistic regression was used by setting C = 1000 with ridge regression (in order to remain in the scikit-learn ecosystem where all logistic regressions are penalized). We also randomly permuted the response variable and refit exactly as in the first-described experiment to produce noise GBC (NGBC).

### Assumptions

It is assumed that the majority of patients in the dataset who have an episode of AKI are, by medical history, high risk for AKI. Conversely, we assume that the majority of patients without AKI have past medical histories that are low risk for AKI. This is paradoxically a strong assumption. To see why, consider a patient with high risk for AKI. We hope to associate this patient’s prior hospitalizations with high risk. Upon rehospitalization, however, suppose that an admitting provider, evaluating the risk as high, decides to administer extra fluids. Ultimately, and fortunately for the patient, this effort may prevent AKI. However, the training set now contains a high risk history coupled to a hospitalization in which *AKI did not occur*. Hence, this patient’s high-risk history will incorrectly be associated with a flipped label of non-AKI. Conversely, a patient with low AKI risk might receive a medication with the potential for causing AKI, resulting in a similar mismatch. It is therefore assumed that the modifications of disease course just described contribute negligible bias to our predictions, but recognized that this bias *is not detectable via internal or external validation*. If this assumption is false, it would invalidate our approach, and future work will focus on developing methods to test this assumption. Notably, this assumption has been shown to fail in a previous study on pneumonia where patients with risk-increasing asthma were given systematic, preferential treatment, effectively flipping their labels [53]. Bias resulting from interventions could be removed by incorporating events that occur during rehospitalization as predictors. However, this is precluded because an intervention could occur all the way up to AKI (e.g., a provider might discontinue intravenous fluids and increase the risk of AKI). Many of our labels are diagnosis codes assigned at the end of the hospitalization, so we do not know when AKI occurred. With the interpretable HPLR1, it is at least possible to confirm that the features are reasonable and appear not to be subject to this bias.

It is also assumed that a time-based (2-year) sample approximates an ideal patient-based sample. Repeating training on a patient-based sample would be a useful complement to this study, and if implemented in the EHR should be formulated as such, since a patient may have a previous hospitalization or rehospitalization outside of the sample. Similarly, it is assumed that our dataset sampled from only one hospital network is representative enough for learning local patterns. We strongly recommend retraining if the model is to be used outside of the population that generated the training data. Finally, it is assumed that undetected AKI from lack of sCr measuremenst or no assignment of a diagnosis code is a rare event.

### Evaluation

For evaluation, 50 iterations of nested (except HP determination, as described above) 5-fold CV were performed. Since any two hospitalizations from the same patient were correlated, CV sampling was “grouped” at the patient level. Micro (over all 250 outer folds) and macro (over 50 iterations) mean and standard deviation of all metrics are reported. As recommended in [49], a probability estimate rather than binary output is provided so the final decision can be made with maximal information at the point of care (e.g., if one patient has 0.499 risk and another has 0.501, these should not be converted to 0 and 1 by an algorithm, but by a provider in clinical context). Although calibration is primarily assessed, discrimination is also described, as is standard practice, with receiver operating characteristic (ROC) and precision-recall (PR) curves and corresponding areas. For calibration, curves are shown with Brier score. *Every* calibration curve shown contains 10 bins. In addition to hospitalization-level performance of GBC, patient-level performance is also analyzed. This is conveyed via scatter plots of the average risk per patient (e.g., a patient with two hospitalizations, one of which had AKI and the other of which did not has 0.5 observed risk) by the average predicted risk. Calibration curves are superimposed for the cases that had 0 or 1 observed risk (all of the hospitalizations and a subset of the patients).

Since algorithms have potential to harm certain subgroups, algorithmic fairness is an active area of research [54, 55]. Here, an error analysis is performed with special focus on the black box GBC, to detect subgroups for which this might be the case. After stratifying by outcome, the same iterated, semi-nested CV procedure described above was used to fit an *l*1-penalized linear regression with either diagnosis, race, gender, or age alone as features and the absolute magnitude of the error as the response (minimum 0, maximum 1). To analyze error by utilization, patients were binned based on the number of hospitalizations that they generated and average error was plotted for each bin. The relationship between number of hospitalizations from a patient and that patient’s impact on coefficients was assessed by removing all hospitalizations from each patient and fitting HPLR1 and then comparing to the coefficients of HPLR1 fit on the full dataset. The comparison was made using *l*1 norm because the coefficient vectors were low dimensional for HPLR1. Error was also assessed as it related to method of diagnosis (code or sCr) and variance of predicted risk.

### Computing environment

All computational work was performed in Python 2.7.14. Libraries in scikit-learn [56–60], keras [61], and the SciPy ecosystem [62–67] were used throughout. Code was run on a linux-based cluster. Each experiment was run via an sbatch script requesting roughly 1 node and 100 to 200 GB of random-access memory. All iterations were distributed using job arrays. Code will be made available upon publication at https://github.com/samuelweisenthal/reh_aki.

## Results

### AKI cohort selection

**Fig 2:**
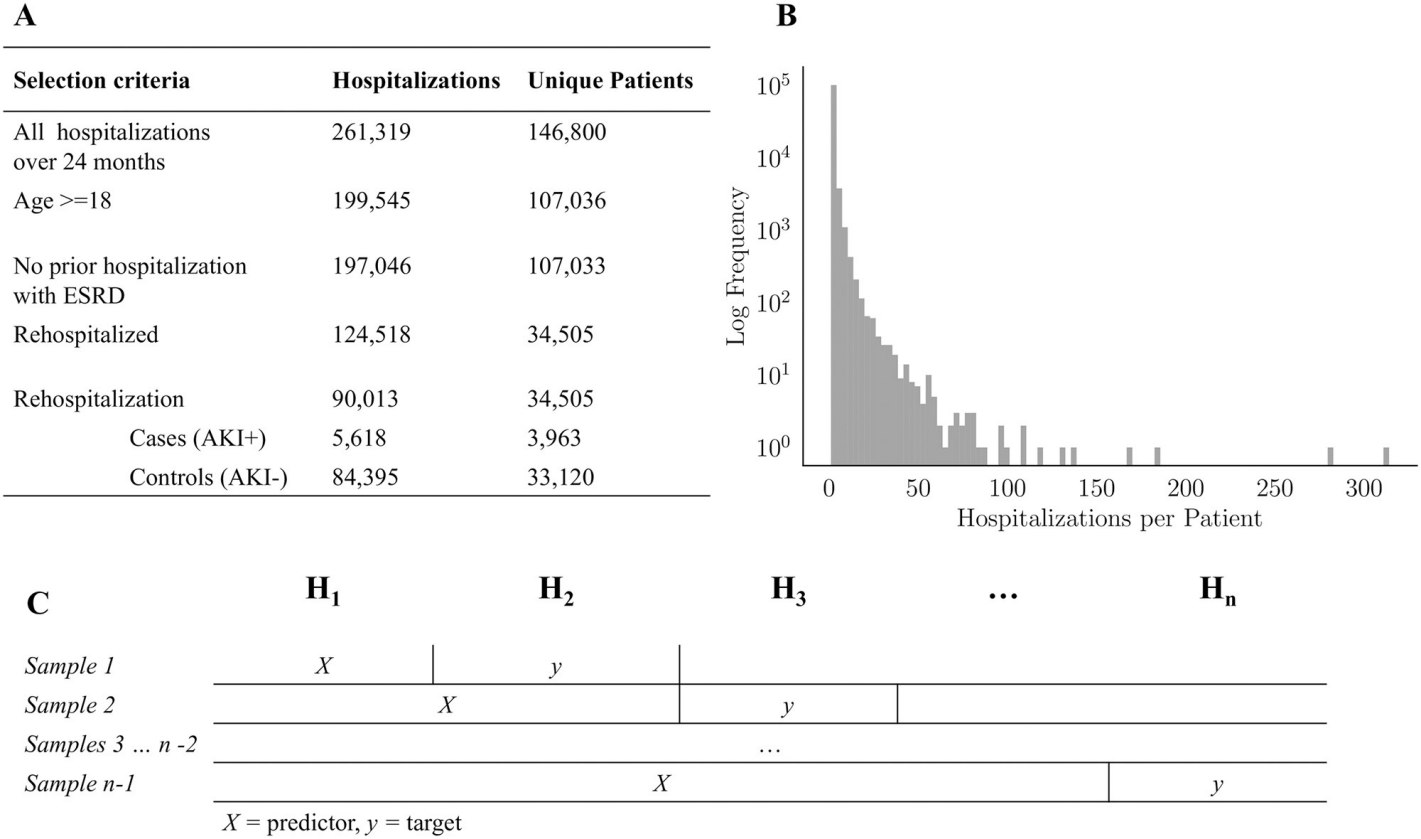
Cohort selection. On the top left, the selection procedure used to obtain the rehospitalization cohort is shown. On the top right, the distribution of the 197,046 hospitalizations not preceded by a diagnosis of ESRD is shown. On the bottom, a schematic of predictor/target generation is shown for an example patient with *n* hospitalizations from which *n* − 1 training cases were derived. For each target rehospitalization, *y*, data from all prior hospitalizations, *X*, are used as predictors. Multiple prior hospitalizations were aggregated using *G* as described above.

A cohort selection schema and results are shown in Fig 2 along with a histogram of the number of hospitalizations per patient. During the two-year window, 146,800 patients generated 261,319 hospitalizations; after excluding hospitalizations with age at admission < 18, 107,036 patients generated 199,545 hospitalizations. Excluding hospitalizations preceded by diagnosis of ESRD, but not preceded by a renal transplant, yielded 197,046 hospitalizations for 107,033 patients. Of these patients, 34,505 (32.2%) were rehospitalized at least once during the two-year period, accounting for 123,828 (62.8%) of total hospitalizations. Within hospitalizations generated by these patients, 90,013 were rehospitalizations (i.e., not the first hospitalization from that patient in our dataset). There were 5,618 (6.2%) cases of AKI. The hospitalization:patient ratio was 1.4 for the cases, and 2.5 for controls. Hence the cases showed more patient-level diversity than the controls, which were generated by patients who returned more often.

### AKI diagnosis

AKI was identified by both diagnosis code and sCr, shown in Table 2. Of all 197,046 hospitalizations in our cohort (not by a patient with previous diagnosed ESRD), 11,166 (5.7%) involved AKI; 4,135 were diagnosed by sCr but not code, 2,747 by sCr and code, and 4,284 by code but not sCr.

**Table 2:** AKI diagnosis distribution.

		**Lab Diagnosis**		**Total**
		**+**	**-**	
**Coding Diagnosis**	**+**	2,747	4,284	7,031
	**-**	4,135	185,880	190,015
**Total**		6,882	190,164	197,046

Cohort demographics for all 124,518 adult hospitalizations (after exclusion of cases following a diagnosis of ESRD) generated by patients who were rehospitalized at some time are shown in Table 3. This corresponds to the fourth cohort shown in Fig 2. These summary statistics are by hospitalization, and not patient, and therefore some patients are represented multiple times. Note that ESRD is present since a hospitalization can contain a diagnosis of ESRD (i.e., permanent kidney failure) even though it does not follow a hospitalization with diagnosis of ESRD. General cohort demographics corresponded to known findings. As expected, hospitalizations in which AKI occurred had higher age on admission [25] and longer duration [3]. A higher proportion of AKI+ subjects were male and white. Also more prevalent in the AKI+ hospitalizations were previously identified risk factors [4, 14, 68] including prior CKD diagnosis [69], prior dialysis procedures without ESRD [69], congestive heart failure [68, 70], diabetes [68], shock [52], and liver failure [71, 72].

**Table 3:** Cohort demographics. Statistics are computed per hospitalization. There are a total of 124,518 hospitalizations from 34,505 patients, each with more than one hospitalization. These are therefore all hospitalizations generated by patients in the final cohort (including the first hospitalization from each patient, for which AKI is not predicted).

Variable	AKI+	(n = 7,762)	AKI-	(n = 116,756)
	**Mean ± STD**	**Median**	**Mean ± STD**	**Median**
**Age**	62.06 ± 17.23	63.00	44.01 ± 18.86	42.00
**Length of Stay**	14.22 ± 22.59	7.89	1.89 ± 4.89	0.33
	**Count**	**%**	**Count**	**%**
**Female**	3,497	44.0	67,811	56.0
**American Indian**	5	0.0	205	0.0
**Asian**	61	1.0	1,364	1.0
**Black**	1,822	23.0	39,038	28.0
**White**	5,557	73.0	68,003	64.0
**Other**	322	4.0	8351	7.0
**Chronic Kidney Disease (CKD)**	2,574	37.0	2,349	2.0
**ESRD**	246	10.0	213	1.0
**Dialysis**	538	15.0	217	1.0
**Renal Transplant**	22	0.0	29	0.0
**Unspecified Renal Failure**	238	3.0	72	0.0
**Congestive Heart Failure**	2,227	29.0	3,218	2.0
**Diabetes**	2,651	34.0	9,031	7.0
**Shock**	997	14.0	2,700	2.0
**Liver Failure**	720	9.0	1,076	1.0
**Rhabdomyolysis**	189	2.0	225	0.0

### Evaluation

**Fig 3:**
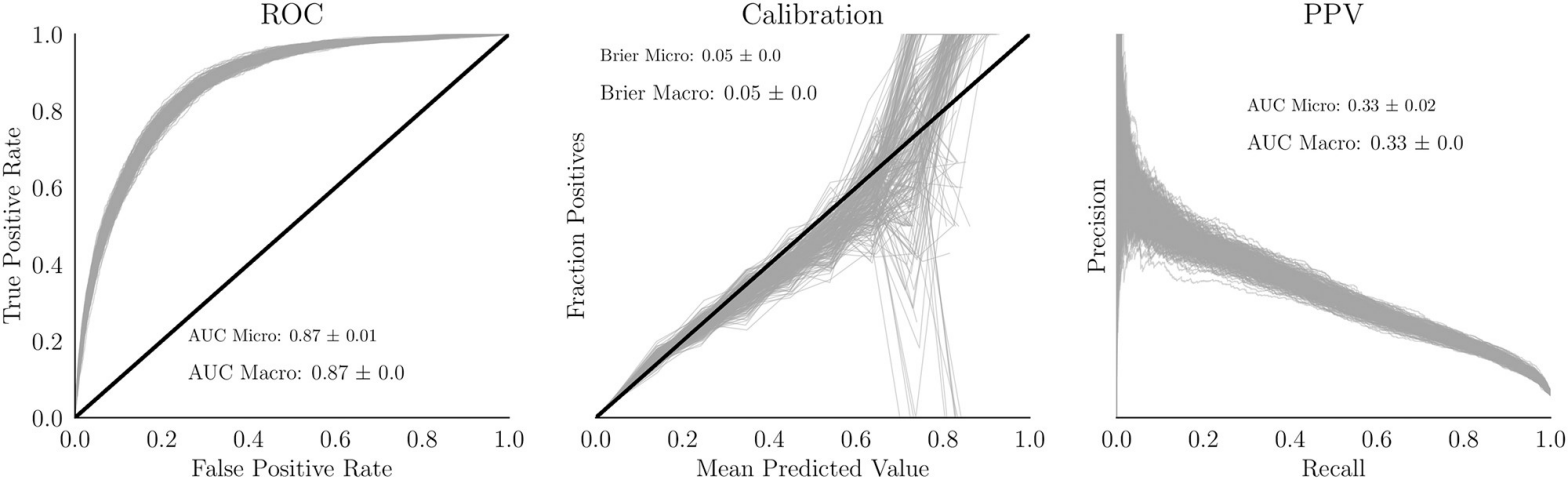
GBC evaluation. ROC, Calibration, and PR curves for 50 iterations of 5-fold CV (250 lines shown; each of 50 iterations has 5 lines corresponding to the 5 outer folds of CV). The black diagonal line represents chance for the ROC curve and ideal for the calibration curve. Results are reported per hospitalization, not patient. Alpha level = 0.5, line weight = 0.5.

The final dataset had 5,308 features at a code precision of 3 digits. After removing features that were observed in fewer than 100 of the samples, 3,387 (63.8%) remained. HP are detailed in Supplement S1 File. All performance metrics are reported in Table 4; since the distributions of these individual metrics were approximately normal (Supplement S1 Fig), standard deviation is reported. Also because of approximate normality, the Bayesian correlated t-test [73] was used to compare systems (Table 5). We specified *a priori* the regions of practical equivalence (ROPE) for ROC AUC, Brier Score, and PR AUC as, respectively, (0.01, 0.001, 0.01). For metric *m* with ROPE *r* and systems in row *i* and column *j*, tuples in the table correspond to (*P*(*m*(*i*) − *m*(*j*)) > 0.5*r*, *P*(*m*(*i*) − *m*(*j*)) ∈ *r*, *P*(*m*(*i*) − *m*(*j*)) < −0.5*r* or, informally, (P(*i* higher score than *j*), P(*i* and *j* practically equivalent), P(*j* higher score than *i*)). Note that ROC and PR are both ideal if 1 and Brier score is ideal if 0, so the Brier table is opposite the other two. We again emphasize that this is a comparison of trained systems, not of the training algorithms, because HP is a confounder. GBC curves are displayed in Fig 3. In the low range, GBC has transposed-sigmoidal tendency suggesting overconfidence (predicting low probabilities as too low and high probabilities as too high). This may be due to dependencies or perhaps a relatively small ratio of cases to features. In contrast to high ROC AUC, precision suffers greatly when the threshold is lowered. PPV is dependent on the prevalence of AKI; even a small false positive rate (FPR) might lead to a high false positive (FP) count if the controls outnumber the cases, as is the case with AKI. Thus even with a low FPR, it can be expected that detecting a TP would cost many FP. FP in AKI, relative to other diseases, are most often fairly innocuous. Preventative measures consist mainly of hydration and medication review. In some cases, however, a FP might result in withholding necessary treatment (e.g., imaging or medication) or unnecessary Nephrology consults [74]. This shortcoming is therefore notable. Ultimately, however, we recommend that a decision based on some threshold *never* be provided to a user in place of a probability estimate [49].

**Table 4:** Predictive performance. ROC = Receiver Operating Characteristic, PR = Precision Recall, ALR1 = Anscombe LR1, GBC = Gradient Boosting Classifier, LR1 = *l*1-Penalized Logistic Regression, LSTM = Long Short-term Memory, HP = Highly Penalized, W = Weighted, S = Sampled, R = Recent (for GBC) or Randomized (for LR1, HPLR1), M = Medication, N = Noise.

		ROC AUC	Brier Score	PR
**GBC**	Micro:	**0.86737 ± 0.00566**	**0.04901 ± 0.00179**	0.32568 ± 0.01502
	Macro:	**0.86737 ± 0.00045**	**0.04901 ± 8e-05**	0.32568 ± 0.0026
**LR1**	Micro:	0.86012 ± 0.00602	0.05038 ± 0.00187	0.30068 ± 0.01533
	Macro:	0.86012 ± 0.00041	0.05038 ± 0.00011	0.30068 ± 0.00182
**ALR1**	Micro:	0.86188 ± 0.00606	0.05019 ± 0.00187	0.30445 ± 0.01571
	Macro:	0.86188 ± 0.00113	0.05019 ± 0.00025	0.30445 ± 0.00411
**RLR1**	Micro:	0.85312 ± 0.00621	0.05068 ± 0.0019	0.30227 ± 0.01453
	Macro:	0.85312 ± 0.00055	0.05068 ± 9e-05	0.30227 ± 0.00159
**HPLR1**	Micro:	0.84545 ± 0.0064	0.05158 ± 0.00191	0.29002 ± 0.01361
	Macro:	0.84545 ± 0.00037	0.05158 ± 5e-05	0.29002 ± 0.00091
**RHPLR1**	Micro:	0.848 ± 0.00651	0.05102 ± 0.00192	0.29869 ± 0.0142
	Macro:	0.848 ± 0.05102	0.05102 ± 8e-05	0.29869 ± 0.00122
**WGBC**	Micro:	0.86328 ± 0.00568	0.04932 ± 0.00178	0.31572 ± 0.01541
	Macro:	0.86328 ± 0.00059	0.04932 ± 0.0001	0.31572 ± 0.00261
**WLR1**	Micro:	0.84965 ± 0.00606	0.0507 ± 0.00188	0.29564 ± 0.01425
	Macro:	0.84965 ± 0.00046	0.0507 ± 6e-05	0.29564 ± 0.00091
**WHPLR1**	Micro:	0.77923 ± 0.01208	0.05387 ± 0.00209	0.25742 ± 0.01609
	Macro:	0.77923 ± 0.00308	0.05387 ± 0.00013	0.25742 ± 0.00385
**SGBC**	Micro:	0.85962 ± 0.00744	0.05326 ± 0.00195	**0.33161 ± 0.02153**
	Macro:	0.85962 ± 0.00226	0.05326 ± 0.00045	**0.33161 ± 0.00631**
**SLR1**	Micro:	0.84752 ± 0.00792	0.05486 ± 0.00206	0.30596 ± 0.02022
	Macro:	0.84752 ± 0.00214	0.05486 ± 0.00049	0.30596 ± 0.00548
**SHPLR1**	Micro:	0.7706 ± 0.01157	0.05754 ± 0.00229	0.28547 ± 0.01992
	Macro:	0.7706 ± 0.00366	0.05754 ± 0.0005	0.28547 ± 0.0054
**RGBC**	Micro:	0.86306 ± 0.00572	0.04927 ± 0.00178	0.32198 ± 0.01526
	Macro:	0.86306 ± 0.00039	0.04927 ± 6e-05	0.32198 ± 0.00185
**MGBC**	Micro:	0.82635 ± 0.00693	0.05161 ± 0.00189	0.27079 ± 0.01484
	Macro:	0.82635 ± 0.00075	0.05161 ± 8e-05	0.27079 ± 0.00172
**MLR1**	Micro:	0.80671 ± 0.00764	0.0564 ± 0.0022	0.22051 ± 0.01397
	Macro:	0.80671 ± 0.00137	0.0564 ± 0.00019	0.22051 ± 0.00212
**LSTM**	Micro:	0.85744 ± 0.00592	0.05027 ± 0.0018	0.28209 ± 0.01547
	Macro:	0.85744 ± 0.0008	0.05027 ± 0.00012	0.28209 ± 0.00526
**CLR**	Micro:	0.80149 ± 0.00785	0.05356 ± 0.00204	0.22926 ± 0.01467
	Macro:	0.80149 ± 0.00034	0.05356 ± 6e-05	0.22926 ± 0.0009
**NGBC**	Micro:	0.49938 ± 0.00837	0.05853 ± 0.0015	0.06251 ± 0.00242
	Macro:	0.49938 ± 0.00399	0.05853 ± 2e-05	0.06251 ± 0.00094

**Table 5:** Predictive performance comparison. ROC = Receiver Operating Characteristic, PR = Precision Recall, ALR1 = Anscombe LR1, GBC = Gradient Boosting Classifier, LR1 = *l*1-Penalized Logistic Regression, LSTM = Long Short-term Memory, HP = Highly Penalized, W = Weighted, S = Sampled, R = Recent (for GBC) or Randomized (for LR1, HPLR1), M = Medication, N = Noise.

**PR**	LR1	ALR1	RLR1	HPLR1	RHPLR1	WGBC	WLR1	WHPLR1	SGBC	SLR1	SHPLR1	RGBG	MGBC	MLR1	LSTM	CLR	NGBC
GBC	1.0, 0.0, 0.0	0.99, 0.01, 0.0	1.0, 0.0, 0.0	1.0, 0.0, 0.0	1.0, 0.0, 0.0	0.49, 0.51, 0.0	1.0, 0.0, 0.0	1.0, 0.0, 0.0	0.06, 0.6, 0.34	0.84, 0.16, 0.0	1.0, 0.0, 0.0	0.07, 0.93, 0.0	1.0, 0.0, 0.0	1.0, 0.0, 0.0	1.0, 0.0, 0.0	1.0, 0.0, 0.0	1.0, 0.0, 0.0
LR1	—————	0.0, 1.0, 0.0	0.0, 0.99, 0.01	0.56, 0.44, 0.0	0.01, 0.99, 0.0	0.0, 0.16, 0.84	0.05, 0.95, 0.0	1.0, 0.0, 0.0	0.0, 0.03, 0.97	0.05, 0.64, 0.31	0.71, 0.29, 0.0	0.0, 0.02, 0.98	1.0, 0.0, 0.0	1.0, 0.0, 0.0	0.91, 0.09, 0.0	1.0, 0.0, 0.0	1.0, 0.0, 0.0
ALR1	—————	—————	0.01, 0.99, 0.0	0.83, 0.17, 0.0	0.14, 0.86, 0.0	0.0, 0.4, 0.6	0.36, 0.64, 0.0	1.0, 0.0, 0.0	0.0, 0.05, 0.95	0.11, 0.71, 0.18	0.82, 0.17, 0.0	0.0, 0.09, 0.91	1.0, 0.0, 0.0	1.0, 0.0, 0.0	0.97, 0.03, 0.0	1.0, 0.0, 0.0	1.0, 0.0, 0.0
RLR1	—————	—————	—————	0.73, 0.27, 0.0	0.01, 0.99, 0.0	0.0, 0.21, 0.79	0.08, 0.92, 0.0	1.0, 0.0, 0.0	0.0, 0.03, 0.97	0.07, 0.68, 0.25	0.77, 0.23, 0.0	0.0, 0.02, 0.98	1.0, 0.0, 0.0	1.0, 0.0, 0.0	0.94, 0.06, 0.0	1.0, 0.0, 0.0	1.0, 0.0, 0.0
HPLR1	—————	—————	—————	—————	0.0, 0.7, 0.3	0.0, 0.0, 1.0	0.0, 0.9, 0.1	1.0, 0.0, 0.0	0.0, 0.0, 1.0	0.0, 0.25, 0.75	0.26, 0.7, 0.04	0.0, 0.0, 1.0	0.96, 0.04, 0.0	1.0, 0.0, 0.0	0.36, 0.64, 0.0	1.0, 0.0, 0.0	1.0, 0.0, 0.0
RHPLR1	—————	—————	—————	—————	—————	0.0, 0.05, 0.95	0.0, 1.0, 0.0	1.0, 0.0, 0.0	0.0, 0.01, 0.99	0.03, 0.59, 0.38	0.64, 0.35, 0.0	0.0, 0.0, 1.0	1.0, 0.0, 0.0	1.0, 0.0, 0.0	0.85, 0.15, 0.0	1.0, 0.0, 0.0	1.0, 0.0, 0.0
WGBC	—————	—————	—————	—————	—————	—————	0.99, 0.01, 0.0	1.0, 0.0, 0.0	0.0, 0.27, 0.72	0.49, 0.49, 0.02	0.98, 0.02, 0.0	0.0, 0.8, 0.2	1.0, 0.0, 0.0	1.0, 0.0, 0.0	1.0, 0.0, 0.0	1.0, 0.0, 0.0	1.0, 0.0, 0.0
WLR1	—————	—————	—————	—————	—————	—————	—————	1.0, 0.0, 0.0	0.0, 0.01, 0.99	0.01, 0.47, 0.51	0.51, 0.48, 0.01	0.0, 0.0, 1.0	1.0, 0.0, 0.0	1.0, 0.0, 0.0	0.71, 0.29, 0.0	1.0, 0.0, 0.0	1.0, 0.0, 0.0
WHPLR1	—————	—————	—————	—————	—————	—————	—————	—————	0.0, 0.0, 1.0	0.0, 0.0, 1.0	0.0, 0.04, 0.96	0.0, 0.0, 1.0	0.0, 0.34, 0.66	1.0, 0.0, 0.0	0.0, 0.05, 0.95	0.98, 0.02, 0.0	1.0, 0.0, 0.0
SGBC	—————	—————	—————	—————	—————	—————	—————	—————	—————	0.99, 0.01, 0.0	1.0, 0.0, 0.0	0.48, 0.49, 0.02	1.0, 0.0, 0.0	1.0, 0.0, 0.0	1.0, 0.0, 0.0	1.0, 0.0, 0.0	1.0, 0.0, 0.0
SLR1	—————	—————	—————	—————	—————	—————	—————	—————	—————	—————	0.97, 0.03, 0.0	0.0, 0.27, 0.73	0.99, 0.01, 0.0	1.0, 0.0, 0.0	0.89, 0.11, 0.0	1.0, 0.0, 0.0	1.0, 0.0, 0.0
SHPLR1	—————	—————	—————	—————	—————	—————	—————	—————	—————	—————	—————	0.0, 0.0, 1.0	0.68, 0.31, 0.01	1.0, 0.0, 0.0	0.26, 0.63, 0.11	1.0, 0.0, 0.0	1.0, 0.0, 0.0
RGBG	—————	—————	—————	—————	—————	—————	—————	—————	—————	—————	—————	—————	1.0, 0.0, 0.0	1.0, 0.0, 0.0	1.0, 0.0, 0.0	1.0, 0.0, 0.0	1.0, 0.0, 0.0
MGBC	—————	—————	—————	—————	—————	—————	—————	—————	—————	—————	—————	—————	—————	1.0, 0.0, 0.0	0.0, 0.41, 0.59	1.0, 0.0, 0.0	1.0, 0.0, 0.0
MLR1	—————	—————	—————	—————	—————	—————	—————	—————	—————	—————	—————	—————	—————	—————	0.0, 0.0, 1.0	0.0, 0.58, 0.42	1.0, 0.0, 0.0
LSTM	—————	—————	—————	—————	—————	—————	—————	—————	—————	—————	—————	—————	—————	—————	—————	1.0, 0.0, 0.0	1.0, 0.0, 0.0
CLR	—————	—————	—————	—————	—————	—————	—————	—————	—————	—————	—————	—————	—————	—————	—————	—————	1.0, 0.0, 0.0
NGBC	—————	—————	—————	—————	—————	—————	—————	—————	—————	—————	—————	—————	—————	—————	—————	—————	—————
**Brier**	LR1	ALR1	RLR1	HPLR1	RHPLR1	WGBC	WLR1	WHPLR1	SGBC	SLR1	SHPLR1	RGBG	MGBC	MLR1	LSTM	CLR	NGBC
GBC	0.0, 0.05, 0.95	0.0, 0.23, 0.77	0.0, 0.0, 1.0	0.0, 0.0, 1.0	0.0, 0.0, 1.0	0.0, 1.0, 0.0	0.0, 0.0, 1.0	0.0, 0.0, 1.0	0.0, 0.0, 1.0	0.0, 0.0, 1.0	0.0, 0.0, 1.0	0.0, 1.0, 0.0	0.0, 0.0, 1.0	0.0, 0.0, 1.0	0.0, 0.13, 0.87	0.0, 0.0, 1.0	0.0, 0.0, 1.0
LR1	—————	0.0, 1.0, 0.0	0.0, 1.0, 0.0	0.0, 0.19, 0.81	0.0, 0.97, 0.03	0.6, 0.4, 0.0	0.0, 1.0, 0.0	0.0, 0.0, 1.0	0.0, 0.03, 0.97	0.0, 0.0, 1.0	0.0, 0.0, 1.0	0.66, 0.34, 0.0	0.0, 0.19, 0.81	0.0, 0.0, 1.0	0.0, 1.0, 0.0	0.0, 0.0, 1.0	0.0, 0.0, 1.0
ALR1	—————	—————	0.0, 1.0, 0.0	0.0, 0.06, 0.94	0.0, 0.79, 0.21	0.3, 0.7, 0.0	0.0, 1.0, 0.0	0.0, 0.0, 1.0	0.0, 0.02, 0.98	0.0, 0.0, 1.0	0.0, 0.0, 1.0	0.38, 0.62, 0.0	0.0, 0.07, 0.93	0.0, 0.0, 1.0	0.0, 1.0, 0.0	0.0, 0.0, 1.0	0.0, 0.0, 1.0
RLR1	—————	—————	—————	0.0, 0.7, 0.3	0.0, 1.0, 0.0	0.96, 0.04, 0.0	0.0, 1.0, 0.0	0.0, 0.0, 1.0	0.0, 0.05, 0.95	0.0, 0.0, 1.0	0.0, 0.0, 1.0	0.96, 0.04, 0.0	0.0, 0.61, 0.39	0.0, 0.0, 1.0	0.01, 0.99, 0.0	0.0, 0.0, 1.0	0.0, 0.0, 1.0
HPLR1	—————	—————	—————	—————	0.0, 1.0, 0.0	1.0, 0.0, 0.0	0.27, 0.73, 0.0	0.0, 0.0, 1.0	0.0, 0.24, 0.76	0.0, 0.01, 0.99	0.0, 0.0, 1.0	1.0, 0.0, 0.0	0.0, 1.0, 0.0	0.0, 0.0, 1.0	0.88, 0.12, 0.0	0.0, 0.0, 1.0	0.0, 0.0, 1.0
RHPLR1	—————	—————	—————	—————	—————	1.0, 0.0, 0.0	0.0, 1.0, 0.0	0.0, 0.0, 1.0	0.0, 0.1, 0.9	0.0, 0.0, 1.0	0.0, 0.0, 1.0	1.0, 0.0, 0.0	0.0, 0.95, 0.05	0.0, 0.0, 1.0	0.18, 0.82, 0.0	0.0, 0.0, 1.0	0.0, 0.0, 1.0
WGBC	—————	—————	—————	—————	—————	—————	0.0, 0.04, 0.96	0.0, 0.0, 1.0	0.0, 0.0, 1.0	0.0, 0.0, 1.0	0.0, 0.0, 1.0	0.0, 1.0, 0.0	0.0, 0.0, 1.0	0.0, 0.0, 1.0	0.0, 0.59, 0.41	0.0, 0.0, 1.0	0.0, 0.0, 1.0
WLR1	—————	—————	—————	—————	—————	—————	—————	0.0, 0.0, 1.0	0.0, 0.05, 0.95	0.0, 0.0, 1.0	0.0, 0.0, 1.0	0.96, 0.04, 0.0	0.0, 0.63, 0.37	0.0, 0.0, 1.0	0.01, 0.99, 0.0	0.0, 0.0, 1.0	0.0, 0.0, 1.0
WHPLR1	—————	—————	—————	—————	—————	—————	—————	—————	0.36, 0.58, 0.06	0.03, 0.47, 0.49	0.0, 0.01, 0.99	1.0, 0.0, 0.0	1.0, 0.0, 0.0	0.0, 0.0, 1.0	1.0, 0.0, 0.0	0.05, 0.95, 0.0	0.0, 0.0, 1.0
SGBC	—————	—————	—————	—————	—————	—————	—————	—————	—————	0.0, 0.03, 0.97	0.0, 0.0, 1.0	1.0, 0.0, 0.0	0.76, 0.24, 0.0	0.0, 0.02, 0.98	0.98, 0.02, 0.0	0.1, 0.64, 0.25	0.0, 0.0, 1.0
SLR1	—————	—————	—————	—————	—————	—————	—————	—————	—————	—————	0.0, 0.0, 1.0	1.0, 0.0, 0.0	0.99, 0.01, 0.0	0.01, 0.3, 0.7	1.0, 0.0, 0.0	0.61, 0.38, 0.02	0.0, 0.02, 0.98
SHPLR1	—————	—————	—————	—————	—————	—————	—————	—————	—————	—————	—————	1.0, 0.0, 0.0	1.0, 0.0, 0.0	0.55, 0.42, 0.03	1.0, 0.0, 0.0	1.0, 0.0, 0.0	0.08, 0.43, 0.5
RGBG	—————	—————	—————	—————	—————	—————	—————	—————	—————	—————	—————	—————	0.0, 0.0, 1.0	0.0, 0.0, 1.0	0.0, 0.51, 0.49	0.0, 0.0, 1.0	0.0, 0.0, 1.0
MGBC	—————	—————	—————	—————	—————	—————	—————	—————	—————	—————	—————	—————	—————	0.0, 0.0, 1.0	0.91, 0.09, 0.0	0.0, 0.0, 1.0	0.0, 0.0, 1.0
MLR1	—————	—————	—————	—————	—————	—————	—————	—————	—————	—————	—————	—————	—————	—————	1.0, 0.0, 0.0	1.0, 0.0, 0.0	0.01, 0.19, 0.8
LSTM	—————	—————	—————	—————	—————	—————	—————	—————	—————	—————	—————	—————	—————	—————	—————	0.0, 0.0, 1.0	0.0, 0.0, 1.0
CLR	—————	—————	—————	—————	—————	—————	—————	—————	—————	—————	—————	—————	—————	—————	—————	—————	0.0, 0.0, 1.0
NGBC	—————	—————	—————	—————	—————	—————	—————	—————	—————	—————	—————	—————	—————	—————	—————	—————	—————
**ROC**	LR1	ALR1	RLR1	HPLR1	RHPLR1	WGBC	WLR1	WHPLR1	SGBC	SLR1	SHPLR1	RGBG	MGBC	MLR1	LSTM	CLR	NGBC
GBC	0.01, 0.99, 0.0	0.0, 1.0, 0.0	1.0, 0.0, 0.0	1.0, 0.0, 0.0	1.0, 0.0, 0.0	0.0, 1.0, 0.0	1.0, 0.0, 0.0	1.0, 0.0, 0.0	0.25, 0.75, 0.0	1.0, 0.0, 0.0	1.0, 0.0, 0.0	0.0, 1.0, 0.0	1.0, 0.0, 0.0	1.0, 0.0, 0.0	0.49, 0.51, 0.0	1.0, 0.0, 0.0	1.0, 0.0, 0.0
LR1	—————	0.0, 1.0, 0.0	0.0, 1.0, 0.0	1.0, 0.0, 0.0	0.91, 0.09, 0.0	0.0, 1.0, 0.0	0.66, 0.34, 0.0	1.0, 0.0, 0.0	0.0, 1.0, 0.0	0.77, 0.23, 0.0	1.0, 0.0, 0.0	0.0, 1.0, 0.0	1.0, 0.0, 0.0	1.0, 0.0, 0.0	0.0, 1.0, 0.0	1.0, 0.0, 0.0	1.0, 0.0, 0.0
ALR1	—————	—————	0.16, 0.84, 0.0	1.0, 0.0, 0.0	0.99, 0.01, 0.0	0.0, 1.0, 0.0	0.95, 0.05, 0.0	1.0, 0.0, 0.0	0.01, 0.99, 0.0	0.89, 0.11, 0.0	1.0, 0.0, 0.0	0.0, 1.0, 0.0	1.0, 0.0, 0.0	1.0, 0.0, 0.0	0.0, 1.0, 0.0	1.0, 0.0, 0.0	1.0, 0.0, 0.0
RLR1	—————	—————	—————	0.03, 0.97, 0.0	0.0, 1.0, 0.0	0.0, 0.45, 0.55	0.0, 1.0, 0.0	1.0, 0.0, 0.0	0.0, 0.85, 0.15	0.1, 0.9, 0.0	1.0, 0.0, 0.0	0.0, 0.51, 0.49	1.0, 0.0, 0.0	1.0, 0.0, 0.0	0.0, 1.0, 0.0	1.0, 0.0, 0.0	1.0, 0.0, 0.0
HPLR1	—————	—————	—————	—————	0.0, 1.0, 0.0	0.0, 0.0, 1.0	0.0, 1.0, 0.0	1.0, 0.0, 0.0	0.0, 0.11, 0.89	0.0, 0.99, 0.01	1.0, 0.0, 0.0	0.0, 0.0, 1.0	1.0, 0.0, 0.0	1.0, 0.0, 0.0	0.0, 0.21, 0.79	1.0, 0.0, 0.0	1.0, 0.0, 0.0
RHPLR1	—————	—————	—————	—————	—————	0.0, 0.0, 1.0	0.0, 1.0, 0.0	1.0, 0.0, 0.0	0.0, 0.32, 0.68	0.0, 0.99, 0.0	1.0, 0.0, 0.0	0.0, 0.01, 0.99	1.0, 0.0, 0.0	1.0, 0.0, 0.0	0.0, 0.59, 0.41	1.0, 0.0, 0.0	1.0, 0.0, 0.0
WGBC	—————	—————	—————	—————	—————	—————	1.0, 0.0, 0.0	1.0, 0.0, 0.0	0.03, 0.97, 0.0	0.94, 0.06, 0.0	1.0, 0.0, 0.0	0.0, 1.0, 0.0	1.0, 0.0, 0.0	1.0, 0.0, 0.0	0.02, 0.98, 0.0	1.0, 0.0, 0.0	1.0, 0.0, 0.0
WLR1	—————	—————	—————	—————	—————	—————	—————	1.0, 0.0, 0.0	0.0, 0.5, 0.5	0.01, 0.99, 0.0	1.0, 0.0, 0.0	0.0, 0.03, 0.97	1.0, 0.0, 0.0	1.0, 0.0, 0.0	0.0, 0.83, 0.17	1.0, 0.0, 0.0	1.0, 0.0, 0.0
WHPLR1	—————	—————	—————	—————	—————	—————	—————	—————	0.0, 0.0, 1.0	0.0, 0.0, 1.0	0.42, 0.58, 0.0	0.0, 0.0, 1.0	0.0, 0.0, 1.0	0.0, 0.0, 1.0	0.0, 0.0, 1.0	0.0, 0.02, 0.98	1.0, 0.0, 0.0
SGBC	—————	—————	—————	—————	—————	—————	—————	—————	—————	0.87, 0.13, 0.0	1.0, 0.0, 0.0	0.0, 0.97, 0.03	1.0, 0.0, 0.0	1.0, 0.0, 0.0	0.02, 0.98, 0.0	1.0, 0.0, 0.0	1.0, 0.0, 0.0
SLR1	—————	—————	—————	—————	—————	—————	—————	—————	—————	—————	1.0, 0.0, 0.0	0.0, 0.07, 0.93	1.0, 0.0, 0.0	1.0, 0.0, 0.0	0.0, 0.51, 0.49	1.0, 0.0, 0.0	1.0, 0.0, 0.0
SHPLR1	—————	—————	—————	—————	—————	—————	—————	—————	—————	—————	—————	0.0, 0.0, 1.0	0.0, 0.0, 1.0	0.0, 0.0, 1.0	0.0, 0.0, 1.0	0.0, 0.0, 1.0	1.0, 0.0, 0.0
RGBG	—————	—————	—————	—————	—————	—————	—————	—————	—————	—————	—————	—————	1.0, 0.0, 0.0	1.0, 0.0, 0.0	0.01, 0.99, 0.0	1.0, 0.0, 0.0	1.0, 0.0, 0.0
MGBC	—————	—————	—————	—————	—————	—————	—————	—————	—————	—————	—————	—————	—————	1.0, 0.0, 0.0	0.0, 0.0, 1.0	1.0, 0.0, 0.0	1.0, 0.0, 0.0
MLR1	—————	—————	—————	—————	—————	—————	—————	—————	—————	—————	—————	—————	—————	—————	0.0, 0.0, 1.0	0.11, 0.89, 0.0	1.0, 0.0, 0.0
LSTM	—————	—————	—————	—————	—————	—————	—————	—————	—————	—————	—————	—————	—————	—————	—————	1.0, 0.0, 0.0	1.0, 0.0, 0.0
CLR	—————	—————	—————	—————	—————	—————	—————	—————	—————	—————	—————	—————	—————	—————	—————	—————	1.0, 0.0, 0.0
NGBC	—————	—————	—————	—————	—————	—————	—————	—————	—————	—————	—————	—————	—————	—————	—————	—————	—————

The distributions of errors by method of diagnosis (i.e., by code or sCr) are shown in Supplement S2 Fig. Without rigorous analysis, it appears that, expectedly, cases detected by both methods have lower mean error than cases detected by one or the other. Notably, cases detected by sCr but not administrative code appear to have higher errors than cases detected by both or cases detected by code but not sCr; this is also to be expected since many cases detected by sCr but not code were likely subtle AKI episodes, or perhaps even correspond to variation in sCr for reasons impossible to discern from the data, but not due to AKI. Gross visual differences between the distributions are not noted, but the slight differences could be an interesting future investigation.

Performance curves for LR1, ALR1, and RLR1 are shown in Supplement S3 Fig, Supplement S4 Fig, and Supplement S5 Fig. Performance curves for HPLR1 and RHPLR1 are shown in Supplement S6 Fig and Supplement S7 Fig. Stability selection included more variables, perhaps since it was less influenced by colinearity. The performance difference between full LR1 and reduced HPLR1 suggests that adjusting for more variables improves, but also increase the variance, of the calibration curves. Performance curves for weighted WGBC are shown in Supplement S8 Fig. When weighting, averaged calibration curves appear to be slightly closer to identity (Supplement S9 Fig). When weighting, performance by utilization, shown in Supplement S10 Fig, appears unchanged. Performance curves for WLR1 and WHPLR1 are shown in Supplement S11 Fig and Supplement S12 Fig, respectively. Performance curves for sampled SGBC, SLR1, and SHPLR1 are shown in Supplement S13 Fig, Supplement S14 Fig, and Supplement S15 Fig. Sampling leads to reduced sample size, and therefore performance appears to generally be worse, but the change is not drastic. Notably, however, PR AUC increases. Performance curves for RGBC, which takes into account only the most recent hospitalization, are shown in Supplement S16 Fig. It is evident that most predictive power is contained in the most recent hospitalization, but a small gain is achieved by including more distant hospitalizations (GBC appears slightly better than RGBC, but the difference is in the region of practical equivalence). Notably, in GBC there are virtually no sums over hospitalizations, only means; when *G* aggregates sequences of hospitalizations of variable length, sums have much higher variance (perhaps why GBC vastly favors labs, which can be converted to less volatile means, while diagnoses are mostly counts). However perhaps when *G* is the identity, such as with RGBC (features not shown), counts are just as well as means. The medication-based performance curves for MGBC and MLR1 are shown respectively in Supplement S17 Fig and Supplement S18 Fig. Performance of CLR is shown in Supplement S19 Fig. This system depends mostly on codes rather than continuous values, probably explaining its reduced performance. Performance of LSTM is shown in Supplement S20 Fig. This system was not exhaustively optimized, so performance is not as strong, but it has the obvious benefit of requiring less feature engineering. Results for NGBC performance, a utilization analysis, and the STD with respect to error are shown in, respectively, Supplement S21 Fig, Supplement S22 Fig, and Supplement S23 Fig. NGBC just predicted 0.06 for every sample.

**Fig 4:**
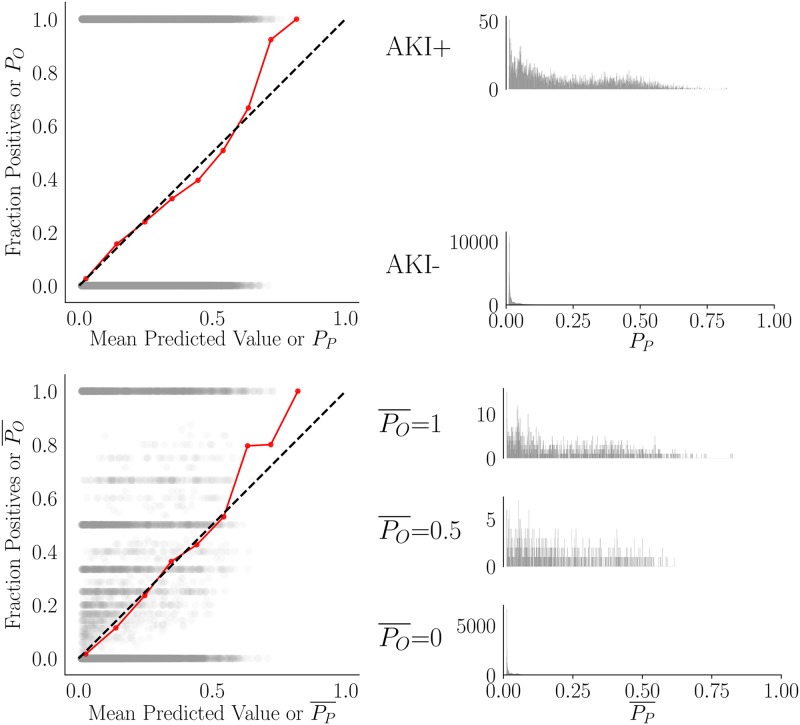
GBC hospitalization- and patient-specific risk distributions. Observed hospitalization-level risk is plotted against predicted risk (top row) and patient-level mean observed risk against mean predicted risk (bottom row). Distributions of predictions *P*_*P*_ are shown at the hospitalization and patient level. At patient level, distributions that are difficult to discern from the scatter plot are shown. In the scatter plots, alpha level is 0.05 and the red calibration curve corresponds to all hospitalizations or to patients who had either mean risk over hospitalizations of 1 or 0. The calibration curves are computed according to the macro-averaged predicted output per hospitalization or patient over the 50 iterations of 5 fold CV (over 250 total folds). Ideal calibration is the dotted black diagonal. Histograms have 1000 bins to give necessary resolution. *P*_*O*_ = observed risk per hospitalization, *P*_*P*_ = predicted risk per hospitalization, $\bar{P_{O}}$ = mean observed risk over hospitalizations, $\bar{P_{P}}$ = mean predicted risk over hospitalizations.

Fig 4 shows the distributions of the probability estimates per hospitalization alongside the same per patient, where the risk is averaged over hospitalizations. Although GBC was not optimized for patient-level prediction, aggregate calibration (averaged over CV folds and trials) appears to be good at the patient level. The calibration curve consisting of averaged predictions is much better than the individual calibration curves per fold. This may be related to the difficulty in sampling each fold at the patient level when there is such a wide variety of hospitalizations per patient. More could be done on characterizing the distributions of the calibration curves. It is apparent from these plots that it is more difficult to predict cases than controls; the distributions of predictions for cases are quite broad and appear almost bimodal.

**Fig 5:**
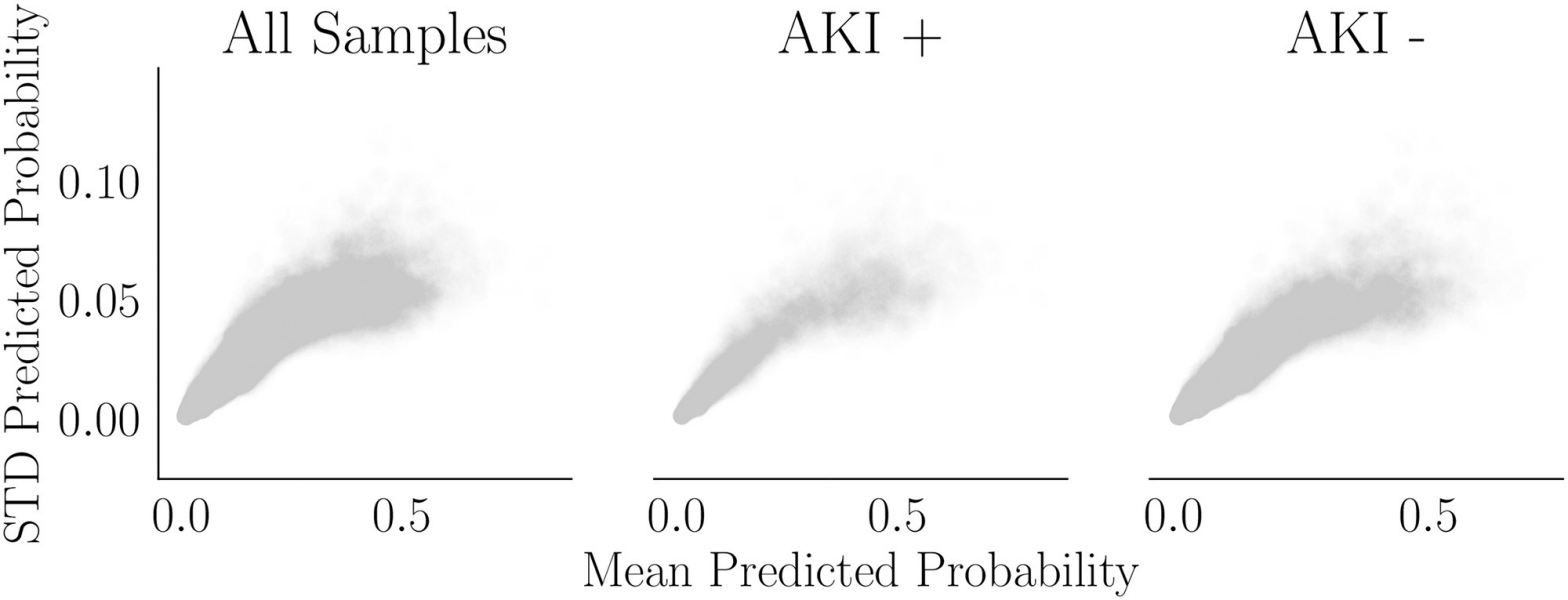
GBC prediction variance. The mean and standard deviation of predicted probabilities are plotted over iterations (per hospitalization). Alpha = 0.01 for all plots.

Uncertainty of predictions appears to increase with increasing predicted risk, even when stratifying by outcome, as shown in Fig 5. Although the range is fairly small (0-0.10), the distributions in Fig 4 show that many of the high risk cases have low predicted risk, so the uncertainty is meaningful. We highlight the necessity of (at least empirical) prediction intervals for GBC, if ever considered for deployment.

### Error analysis of GBC

We sought to identify specific subgroups which are easily recognized by a provider and for which the best performing GBC might make errors. To show that the linear regressions used for this purpose were well fit, train, validation, and test MSE from the fold used to set HP is provided in Supplement S1 Table. HP are provided as well, chosen manually as in the main study by making the train-validation difference in a single fold small, although usually a penalty was not necessary. The mean and standard deviations of nonzero coefficients are shown in Table 6. The diagnoses associated with increased error in the cases (failure to predict AKI when it occurred) are assigned to patients who were hospitalized for reasons not directly related to the kidney (substance abuse). Conversely, the diagnoses associated with small errors are obviously associated with AKI (e.g., we see especially accurate predictions of high AKI risk in hospitalizations preceded by frequent AKI or CKD). The diagnoses associated with increased error in the controls (failure to rule out AKI when it did not occur) were, expectedly, AKI, CKD, and anemia. This is not as revelatory as the cases; GBC has learned that prior kidney disease is associated with future kidney disease, which is a well known phenomenon. These may correspond to cases in which interventions occurred for high risk patients (the label flipping mentioned in Assumption (1)).

**Table 6:** Coefficients of features associated with error. For diagnoses, features correspond to the count assigned in prior hospitalizations. Note that age and diagnosis were fit in separate regressions despite being displayed in the same table.

**Cases (AKI +)**	**Diagnosis**	**Mean (95% CI)**
Lasso (+)	“Non-present” Dx	0.0071 (0.0069, 0.0073)
	Non-dependent abuse of drugs	0.0030 (0.0028, 0.0032)
Lasso (-)	AKI	-0.0476 (-0.0479, -0.0473)
	CKD	-0.0301 (-0.0304, -0.0297)
	Other and unspecified anemias	-0.0066 (-0.0068, -0.0063)
	Convalescence and palliative care	-0.0039 (-0.0042, -0.0036)
	Hypertensive chronic kidney disease	-0.0006 (-0.0008, -0.0004)
	Heart failure	-0.0003 (-0.0003, -0.0002)
	Cardiac dysrhythmias	-0.0001 (-0.0002, -0.0001)
Age		-0.0271 (-0.0272, -0.0269)
**Controls (AKI -)**	**Diagnosis**	**Mean (95% CI)**
Lasso (+)	AKI	0.0166 (0.0164, 0.0167)
	CKD	0.0112 (0.0111, 0.0113)
	Other and unspecified anemias	0.0014 (0.0014, 0.0015)
Age		0.0236 (0.0235, 0.0236)

There were no detected relationships to the error (all coefficients were 0) for different races (American Indian, Asian, Black, Black/American Indian, Declined, Other, Unknown, and White). This is very comforting, although it is difficult to make a general conclusion for the rare races (see Fig 3 for frequencies). Gender also, favorably, showed no relationship to error. As shown in Table 6, increasing age leads to lower error in the cases and higher error in the controls. Hence errors occur because predicted risk is sometimes too high in older patients when they are healthy and too low in younger patients when they are not. Age is a particularly well-recorded variable; it is unclear what variable could be adjusted for to remove this bias, but it is likely that explicit stratification might be in order. In this large sample, the healthy young simply overwhelm the high risk young and opposite for the older patients. A plot of error by age is shown in Supplement S24 Fig to complement the findings in Table 6. It is likely that, at least in part, the correlation of the errors with features indicates slight underfitting; had higher capacity HP been permitted, these patterns might have been detected (at the risk of overfitting in other ways). As described above, bias was prioritized above variance in order to avoid overfitting, but now this error analysis gives some insight into who might suffer from poor predictions as a result.

**Fig 6:**
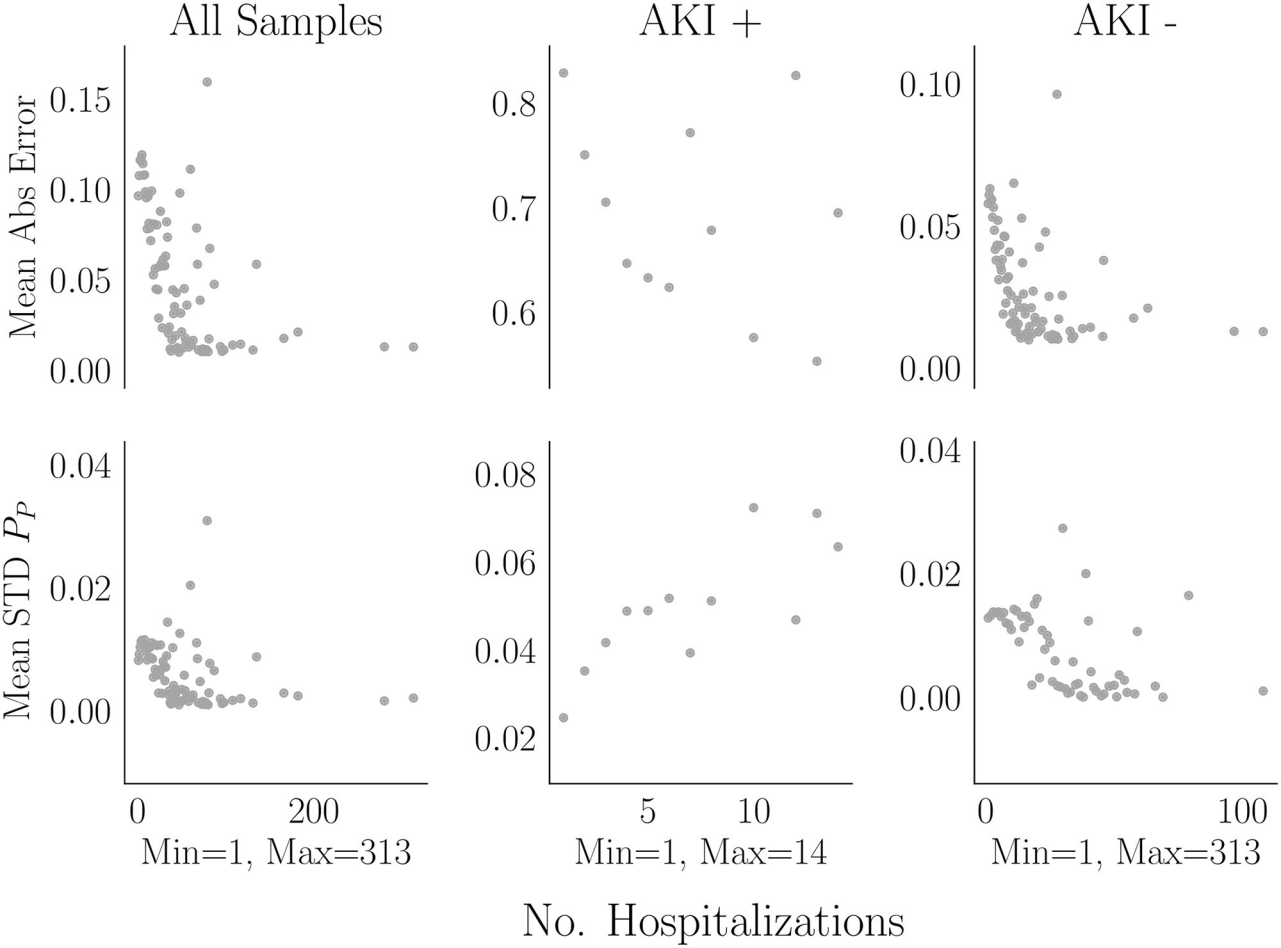
GBC error by utilization. The mean and STD absolute error is shown as a function of the number of hospitalizations. Patients were binned based on the number of hospitalizations in the dataset and then, over bins, the mean error and STD of the predictions were computed. Stratification by outcome is performed since it was earlier established that the hospitalization:patient ratio is higher in cases than in controls.

Error and STD of predicted probability against utilization is shown in Fig 6. The average error for controls decreases with the utilization. For cases, the pattern is not clear, but it also appears to decrease. Hence predictions are better for patients with many hospitalizations. STD however appears to increase with utilization for cases, unlike for controls. Since this dataset is a time-window sample, high utilizers are overrepresented (recall that a patient with multiple hospitalizations appears multiple times in the dataset). This is common in medical prediction problems (e.g., [16] had a final analysis cohort with roughly 1.6 million admissions generated by roughly 600,000 patients; a readmission study [75] had roughly 3.3 million admissions generated by 1.3 million patients). We hypothesize that high utilizers have strong influence over parameters. Consider two patients without AKI; one is hospitalized 10 times and each time merely visits the emergency department and another is hospitalized twice for heart failure exacerbation. The patient with 10 hospitalizations generates 9 training examples while the one with heart failure exacerbation generates only one. The former will have much stronger influence over coefficients.

The impact of each patient on the coefficient vector of HPLR1 is shown in Supplement S25 Fig. There are patients who are relatively high and relatively low utilizers who have substantial impact on the coefficients. Since there are many low utilizers, perhaps there is greater probability that there is a very different patient that might influence coefficients more. However, extreme influencers seem to be relatively high utilizers. Although this optimizes hospitalization-level performance (a prediction error for the patient who generates 10 hospitalizations may lead to 10 errors, while an error for the patient who generates 1 will only result in one, all else being equal) this might not be fair. In WGBC, we have downweighted hospitalizations from high utilizers such that the 9 training examples have a net effect on the coefficients equal to the 1 training example. Making patient influence over coefficients more equal optimizes performance on the level of patients rather than hospitalizations, but we do not see a clear change in the utilization analysis. Another option for future work might be mixed effects approaches.

### Features

Predictors specific to rehospitalized patients are described. Note that these should be considered predictors, not risk factors, since causality is never established. Further, many of the features are correlated, so it is important to note variance. It is possible that some features in the ensuing tables might have correlated counterparts that could just as well have been selected in their places. We still maintain that these tables are useful (1) to demonstrate that the systems depend on reasonable predictors and (2) to report potential predictors for specification of a more parsimonious system that could be validated on a different dataset. Both of these objectives can be met despite the correlated nature of the features. Also, note that the relationships between these features and AKI are associative, not necessarily causal. The distribution of feature importances/coefficients was very skewed and we believe the interesting ones are adequately contained in the top 40, but this cutoff is still arbitrary. With these caveats in mind, we discuss some interesting findings. We reported 95% bootstrap [76] confidence intervals (10,000 iterations using [77]) instead of standard deviation as we had with the metrics because it was difficult to check each coefficient’s distribution for symmetry. Importance scores were computed via scikit-learn according to the Gini importance definition in [78].

#### GBC and LR1

The 40 features with the highest micro-averaged GBC importance scores and largest absolute LR1 coefficients are shown in Table 7 and RLR1 coefficients are shown in Supplement S2 Table. Some features were comprised of sub-features (e.g., diagnosis codes contained many sub-diagnoses). For display in tables, these were succinctly renamed via a representative term (e.g., diuretics or CKD), most frequent sub-features, or general group names from [79].

**Table 7:** Feature importances/coefficients for GBC and LR1. For laboratory results, the first function is *G*, aggregation over hospitalizations, and the second is *F*, aggregation within a hospitalization; e.g., “mean max sCr” is the mean over hospitalizations of the maximum sCr of each hospitalization.

**GBC**	**Mean (95% CI)**
Age	0.0409 (0.0404, 0.0414)
Mean count abnormally high urea nitrogen	0.0351 (0.0338, 0.0365)
Count “non-present” DRGs	0.0295 (0.0289, 0.0301)
Count Dx: AKI	0.0168 (0.0161, 0.0175)
Mean count abnormally low hemoglobin	0.0160 (0.0152, 0.0168)
Mean sum urea nitrogen	0.0153 (0.0144, 0.0162)
Mean sum sCr	0.0143 (0.0135, 0.0151)
Mean min direct bilirubin	0.0139 (0.013, 0.0149)
Mean max albumin	0.0128 (0.0117, 0.0139)
Count immunosuppressant medications	0.0124 (0.0116, 0.0132)
Max mean urea nitrogen	0.0118 (0.0109, 0.0127)
Mean count abnormally high sCr	0.0113 (0.0106, 0.012)
Count pharm subclass: Loop diuretics	0.0104 (0.0098, 0.011)
Count pharm subclass: K-sparing diuretics	0.0099 (0.0089, 0.0108)
Min min direct bilirubin	0.0098 (0.0088, 0.0107)
Mean count abnormal glomerular filtration rate-caucasian	0.0088 (0.0082, 0.0094)
Count “non-present” Dx	0.0084 (0.0078, 0.009)
Min mean chloride	0.0080 (0.0074, 0.0087)
Count “non-present” CPT4 Px	0.0078 (0.0072, 0.0084)
Max max sCr	0.0078 (0.0071, 0.0084)
Mean max urea nitrogen	0.0073 (0.0065, 0.008)
Mean mean hemoglobin	0.0071 (0.0065, 0.0077)
Count Px: injection of glucagon, haloperidol, heparin, enoxaparin	0.0070 (0.0065, 0.0076)
Spironolactone	0.0067 (0.0058, 0.0076)
Count discharges to Hospice/Medical Facility	0.0066 (0.0062, 0.0071)
Count Dx: artificial opening status (e.g., tracheostomy)	0.0065 (0.0058, 0.0072)
Min max albumin	0.0065 (0.0058, 0.0071)
Var count abnormally high urea nitrogen	0.0057 (0.0049, 0.0065)
Count allopurinol	0.0056 (0.0049, 0.0062)
Min min Glomerular filtration rate-Black	0.0055 (0.0049, 0.0061)
Count carbapenems	0.0055 (0.0048, 0.0062)
Spinal procedures w/o CC/MCC	0.0053 (0.0044, 0.0061)
Sum max glomerular filtration rate-Black	0.0052 (0.0047, 0.0057)
Max min sCr	0.0052 (0.0047, 0.0058)
Count Dx: Disorders of fluid electrolyte and acid-base balance	0.0052 (0.0047, 0.0057)
Mean sum glomerular filtration rate-Black	0.0052 (0.0045, 0.0059)
Count Dx: Diabetes Mellitus	0.0051 (0.0046, 0.0056)
Max max urea nitrogen	0.0051 (0.0045, 0.0058)
Max max hemoglobin	0.0049 (0.0043, 0.0055)
Sum max hemoglobin	0.0048 (0.0042, 0.0054)
**LR1 (+)**	**Mean (95% CI)**
Age	0.5846 (0.5825, 0.5867)
Mean mean urea nitrogen	0.1127 (0.108, 0.1175)
Count Dx: AKI	0.0974 (0.0956, 0.0993)
Mean max glucose	0.0905 (0.0882, 0.0927)
Mean mean sCr	0.0587 (0.0532, 0.0641)
Gender: Male	0.0505 (0.0455, 0.0554)
Mean var glomerular filtration rate-Caucasian	0.0442 (0.0424, 0.046)
Max mean sCr	0.0386 (0.0337, 0.0433)
Count discharges with home health organization care services	0.0290 (0.0271, 0.0309)
Max mean urea nitrogen	0.0251 (0.0215, 0.0286)
Count Dx: Chronic pulmonary heart disease	0.0241 (0.0228, 0.0254)
Min min direct bilirubin	0.0241 (0.0221, 0.0261)
Mean min direct bilirubin	0.0227 (0.0208, 0.0245)
Count DRG: Hepatobiliary diagnostic procedures with MCC	0.0213 (0.0196, 0.0229)
Count Px: Pathology consult	0.0195 (0.0181, 0.021)
Count Px: Assay of blood lipoprotein or of magnesium	0.0186 (0.0161, 0.021)
Mean max urea nitrogen	0.0182 (0.015, 0.0213)
Count Px: Assay of urine sodium	0.0179 (0.0163, 0.0194)
Min min sCr	0.0173 (0.0144, 0.0201)
Last primary insurance: other	0.0171 (0.0158, 0.0185)
**LR1 (-)**	**Mean (95% CI)**
Count “non-present” DRGs	-0.2126 (-0.2294, -0.1964)
Mean min albumin	-0.2122 (-0.2171, -0.2076)
Mean min albumin	-0.0623 (-0.0671, -0.0575)
Gender: Female	-0.0610 (-0.0659, -0.0561)
Count Location: High risk labor and delivery unit	-0.0585 (-0.0613, -0.0558)
Count location: Emergency Department	-0.0526 (-0.0606, -0.0443)
Min min glomerular filtration rate-Caucasian	-0.0409 (-0.0446, -0.0373)
Mean min chloride	-0.0393 (-0.0418, -0.0369)
Count Dx: Traumatic injuries	-0.0331 (-0.036, -0.0301)
Mean mean albumin	-0.0307 (-0.0347, -0.0263)
Count Admission on Tuesday	-0.0288 (-0.033, -0.0246)
Count Admission on Saturday	-0.0265 (-0.0307, -0.0221)
Count Dx: Injury from athletics	-0.0252 (-0.0284, -0.0219)
Count discharges on Sunday	-0.0202 (-0.0237, -0.0166)
Mean min glomerular filtration rate-Caucasian	-0.0195 (-0.0229, -0.016)
Max min hemoglobin	-0.0166 (-0.0202, -0.0128)
Min min albumin	-0.0158 (-0.0187, -0.0129)
Max min bicarbonate	-0.0109 (-0.0126, -0.0091)
Mean max albumin	-0.0108 (-0.0128, -0.0088)
Mean min bicarbonate	-0.0105 (-0.0122, -0.0087)

For GBC, many features correspond to known indicators of acute or chronic kidney dysfunction (e.g. diagnosis of AKI, sCr, UN, GFR). As our features are gleaned from prior hospitalizations, they suggest that prior acute or chronic kidney disease increases the probability of AKI. Age is associated with declining kidney function in general, as well as a higher incidence of CKD and other conditions strongly associated with renal disease. Thus it is not surprising that age is the strongest predictor of AKI in both GBC and LR1. Another constellation of highly ranked features carries strong secondary association with underlying kidney disease. These include medications used to treat consequences of decreased kidney function such as allopurinol, used to treat elevated uric acid levels, and loop diuretics, used to reduce fluid retention, edema, and hypertension. Highly ranked features associated with the presence of liver disease (bilirubin) and associated treatment for both liver and heart disease (spironolactone) were also identified. Moderate to advanced liver and heart disease are associated with hepatorenal and cardiorenal syndromes, respectively, with resulting AKI (we even see hepatobiliary diagnostic procedures associated with increased risk in LR1). Hemoglobin is also identified, likely as an indicator of anemia resulting from renal pathology. Interestingly, UN is often slightly preferred to sCr here, perhaps reflecting loss of muscle mass due to catabolism during illness, with an associated lower creatinine production blunting rise in sCr. UN is generally correlated with sCr, probably explaining the high STD in the importances of both.

The LR1 coefficients reveal the sign of predictors. A number of features were associated with lower probability of AKI by LR1, especially those generally associated with populations having a lower incidence of kidney disease, including locations (labor and delivery, emergency department), and diagnoses (injuries from trauma and athletics). Interestingly, although with small coefficients, timing of discharge and admission was identified as predictive. For example, prior Sunday discharge was associated with a lower probability of AKI. This may be due to the common practice in nursing homes and rehabilitation facilities to not accept weekend transfers, giving complicated patients with a higher likelihood of AKI lower probability of Sunday discharge. In contrast, weekend hospital admissions (Saturday admission) have a higher number of traumatic injuries [80] and thus a lower number of conditions associated with AKI (we see that diagnosis of traumatic injury is also present as a negative predictor). In both GBC and LR1, “non-present” diagnoses and procedures were highly ranked since history of few diagnoses and procedures reflect robust health.

Although UN and sCr would likely have been chosen to predict AKI, many of the features studied here are novel representations. For example, rather than just a recent UN, we include the number of abnormal lab flags for UN; rather than just an at-admission sCr, we include the mean over hospitalizations of the sum of sCr per hospitalization; rather than just presence of a loop diuretic on a medication list, we include the actual number of administrations. Features of this form would probably not have been collected *a priori* for AKI prediction, and their components are generally hidden to providers. Many highly ranked features further depend on *the behavior of providers*. This might suggest that to optimize EHR data it is important to capture features that showcase provider behavior–such as testing or prescribing frequency. Commonly used features such as “does this patient have comorbidity X” might be better reformulated as “how many times in this patient’s history has a provider assigned a code for comorbidity X”. The features are further enhanced by EHR-based analyses (abnormal lab flagging).

Interestingly, features associated with AKI in prior studies that analyzed only data available at admission were *not* necessarily detected as the best predictors here in *rehospitalized* patients. For example, laboratory values dominated diagnosis codes, with the exception of diagnoses related to CKD or AKI. We hypothesize that this may be due to our focus on longitudinal measurements, inclusion of more candidate features, the sparsity of ICD-9 codes, or perhaps correlation of diagnoses with laboratory predictors (the latter provide more predictive information, being continuous-valued and reliably collected variables). Laboratory features may also have been boosted by the basis functions *F*, while the codes were generally just counted.

#### HPLR1

All features with nonzero coefficients for HPLR1 are shown in Table 8 and the same for RHPLR1 is shown in Supplement S3 Table. HPLR1 is especially interpretable. UN has a large positive coefficient (note that there are two that are likely correlated and hence have high STD). High glucose (endocrine or metabolic disorders) and potassium (renal dysfunction) are also predictive along with discharge with assisted care (Home Health Org.). Negative coefficients are on mean *minimum* hemoglobin, albumin, and calcium (all resounding laboratory indicators of strong health and robust kidney function). Note that every positive laboratory coefficient contains a maximum and every negative a minimum.

**Table 8:** Coefficients of HPLR1.

HPLR1 Status	Coefficient	Mean (95% CI)
**HPLR1 (+)**	Age	0.2304 (0.229, 0.2318)
	Max max urea nitrogen	0.1752 (0.1695, 0.1811)
	Mean max urea nitrogen	0.1297 (0.1242, 0.1352)
	Count Dx: AKI	0.0248 (0.0236, 0.026)
	Mean max glucose	0.0001 (-0.0, 0.0002)
	Mean max potassium	0.0001 (-0.0, 0.0002)
**HPLR1 (-)**	Mean min hemoglobin	-0.0931 (-0.0949, -0.0912)
	Mean min albumin	-0.0557 (-0.0573, -0.0541)
	Mean min calcium	-0.0001 (-0.0002, 0.0)

### Comparison with features from Cronin et al. [16]

We can compare our features to those in Cronin et al. [16], where a random forest was used to predict AKI stage 1+ (KDIGO stages 1, 2, or 3). In Cronin et al, we see strong dependence on renal indicators (e.g., GFR, UN), labs indirectly associated with renal function (Hemoglobin), heart failure, diuretics (loop, thiazides), and anti-hypertensives such as angiotensin-converting enzyme inhibitors (ACEi), which is also reflected in our findings. Although it is difficult to test rigorously, our study might suggest an opportunity to more extensively incorporate laboratory values from the past as predictors; Cronin et al. only used diagnoses and body mass index further than 365 days back and medications and temperature further than 90 days back.

We can also compare our LR1 with lasso results from Cronin et al. High odds ratios in Cronin et al. were present in patients on antihypertensives (ACEi, angiotensin II receptor blockers, thiazides, *β*-blockers), diagnoses associated with AKI (diabetes, anemia, hyper and hypotension, peripheral vascular disease, HIV, cancer, and rheumatoid arthritis), labs associated with renal function or injury (calcium, hemoglobin, GFR, troponin, bilirubin), and antiobiotics (Sulfa). Again, we see many features associated with renal function, renal medications, sepsis, or cardiovascular dysfunction, which is also reflected in our findings. In our features, but not in Cronin et al., we see discharge to home with outpatient care provided by a home health care organization (e.g. visiting nurse, home physical therapy, home health aide), lab values involving glucose, presence in the high risk labor and delivery unit or in the emergency department, injury from athletics, assay of urine sodium, discharge with organization care services, and marital status (possibly a proxy for age).

#### MGBC & MLR1

A substantial percentage of AKI is due to, or exacerbated by, medications. We were thus interested in examining the medications in prior hospitalizations that might be associated with AKI in subsequent hospitalizations. There were 927 medications analyzed. The most important medication predictors are shown in Table 9. Here again, the combination of GBC and LR1 results is useful to put the identified features in context. Medications used to treat chronic obstructive pulmonary disease, such as albuterol and betamethasone, psychiatric conditions (respiridone, trazadone, aripiprazole), or obstetric therapies (magnesium, pre-natal vitamins) had a negative association with AKI. Our aim was to detect potentially modifiable risk factors, but it is very difficult to disentangle confounders (e.g., Heparin might be associated with thrombotic event prophylaxis, dextrose with diabetic ketoacidosis and malnutrition). Most medications associated with high risk were actually given to protect the kidney and most medications associated with low risk were given in the context of robust kidney health. This analysis might be enhanced by somehow incorporating predictors from the current hospitalization. We re-emphasize that no causal inference can be performed in this study, but interesting findings include tacrolimus (known nephrotoxicity [81]), midazolam (this association has been shown relative to propofol [82]), and oxycodone (opioid nephrotoxicity is currently researched [83]). It is worth highlighting the counter-intuitive finding that ibuprofen administration in prior hospitalizations is a negative predictor for AKI. Probably this is because non-steroidal anti-inflammatory medications (i.e. ibuprofen, ketorolac) are contraindicated in patients with elevated AKI risk, and thus administration during a prior hospitalization is a clinical indicator for low AKI risk. However, patients with extensive histories of ibuprofen use, given its potentially deleterious effect on the kidney, should be monitored *more closely* for AKI. Here however we analyze *administrations*, which, unlike use, reflect provider behavior.

**Table 9:** Feature importances/coefficients for MGBC and MLR1. Each feature corresponds to the count of administrations of the medication over prior hospitalizations.

**MGBC**	**Mean (95% CI)**
Furosemide	0.0718 (0.0706, 0.0731)
Ibuprofen	0.0356 (0.035, 0.0361)
Sodium Chloride	0.0306 (0.0299, 0.0313)
Allopurinol	0.0245 (0.0239, 0.025)
Amlodipine Besylate	0.0237 (0.0232, 0.0242)
Oxycodone-Acetaminophen	0.0237 (0.023, 0.0243)
Spironolactone	0.0232 (0.0224, 0.024)
Heparin Sodium	0.0229 (0.0223, 0.0235)
Tacrolimus	0.0217 (0.0212, 0.0223)
Enoxaparin Sodium	0.0210 (0.0203, 0.0217)
Torsemide	0.0189 (0.0183, 0.0195)
Aspirin	0.0187 (0.0179, 0.0195)
Fentanyl Citrate	0.0183 (0.0171, 0.0194)
Dextrose	0.0180 (0.0175, 0.0186)
Levothyroxine Sodium	0.0154 (0.0148, 0.0161)
Piperacillin-Tazobactam In D	0.0145 (0.014, 0.0151)
Epoetin Alfa	0.0144 (0.0139, 0.0149)
Carvedilol	0.0133 (0.0125, 0.0141)
Hydralazine HCL	0.0128 (0.0121, 0.0135)
Sevelamer Carbonate	0.0119 (0.0111, 0.0126)
Metoprolol Tartrate	0.0113 (0.0107, 0.0118)
Docusate Sodium	0.0111 (0.0103, 0.0119)
Ceftriaxone Sodium In Dextrose	0.0104 (0.0097, 0.0111)
Pantoprazole Sodium	0.0103 (0.0095, 0.0111)
Metformin Hcl	0.0102 (0.0096, 0.0109)
Albuterol Sulfate Hfa	0.0099 (0.0092, 0.0105)
Vancomycin Hcl In Dextrose	0.0099 (0.0092, 0.0105)
Nephro-Vite	0.0090 (0.0084, 0.0096)
Magnesium Sulfate	0.0083 (0.0077, 0.0088)
Midazolam (Versed)	0.0083 (0.0076, 0.0091)
Glycopyrrolate	0.0081 (0.0072, 0.0088)
Paricalcitol	0.0071 (0.0062, 0.0081)
Cyclosporine Modified	0.0070 (0.0061, 0.0079)
Acetaminophen	0.0070 (0.0063, 0.0077)
Benazepril HCL	0.0068 (0.006, 0.0076)
Diltiazem HCL Er Beads	0.0067 (0.0057, 0.0076)
Labetalol HCL	0.0067 (0.006, 0.0073)
Losartan Potassium	0.0064 (0.0058, 0.0071)
Warfarin Sodium	0.0064 (0.0058, 0.007)
Albumin Human	0.0063 (0.0056, 0.0071)
**MLR1 (+)**	**Mean (95% CI)**
Furosemide	0.1643 (0.161, 0.1677)
Heparin Sodium	0.1349 (0.1325, 0.1372)
Allopurinol	0.0947 (0.0912, 0.0982)
Enoxaparin Sodium	0.0883 (0.0857, 0.0911)
Piperacillin-Tazobactam In D	0.0809 (0.079, 0.0828)
Dextrose	0.0785 (0.0725, 0.0844)
Tacrolimus	0.0727 (0.0707, 0.0746)
Metoprolol Tartrate	0.0706 (0.0683, 0.0729)
Hydralazine HCL	0.0668 (0.0647, 0.0689)
Torsemide	0.0609 (0.0583, 0.0637)
Glucagon HCL (Rdna)	0.0545 (0.0483, 0.0606)
Ceftriaxone Sodium In Dextrose	0.0536 (0.0512, 0.056)
Epoetin Alfa	0.0531 (0.051, 0.0553)
Spironolactone	0.0530 (0.0501, 0.056)
Metoprolol Succinate	0.0500 (0.0483, 0.0517)
Sodium Chloride	0.0436 (0.0398, 0.0474)
Moxifloxacin HCL	0.0358 (0.0341, 0.0375)
Ciprofloxacin HCL	0.0347 (0.0326, 0.0368)
Fish Oil	0.0284 (0.0269, 0.0299)
Oxycodone HCL	0.0281 (0.026, 0.0303)
**MLR1 (-)**	**Mean (95% CI)**
Ibuprofen	-0.2765 (-0.2807, -0.2722)
Oxycodone-Acetaminophen	-0.1575 (-0.1607, -0.1544)
Promethazine HCL	-0.0914 (-0.0949, -0.0879)
Ondansetron	-0.0791 (-0.082, -0.0761)
Hydroxyzine Pamoate	-0.0707 (-0.0733, -0.0681)
Albuterol Sulfate Hfa	-0.0576 (-0.0609, -0.0543)
Nicotine Polacrilex	-0.0468 (-0.0501, -0.0434)
Tetanus-Diphth-Acell Pert	-0.0393 (-0.0415, -0.0372)
Cyclobenzaprine HCL	-0.0313 (-0.0335, -0.0292)
Classic Prenatal Vitamin	-0.0312 (-0.0334, -0.0289)
Trazodone HCL	-0.0300 (-0.0331, -0.0268)
Oxytocin	-0.0288 (-0.0309, -0.0267)
Risperidone Microspheres	-0.0264 (-0.0289, -0.024)
Risperidone	-0.0231 (-0.0255, -0.0207)
Lorazepam (Ativan)	-0.0207 (-0.0227, -0.0186)
Ketorolac Tromethamine	-0.0207 (-0.0229, -0.0183)
Betamethasone Acetate & Sodium Phosphate	-0.0119 (-0.0134, -0.0103)
Prenavite Protein Coated	-0.0091 (-0.0116, -0.0064)
Aripiprazole	-0.0067 (-0.008, -0.0053)
Etomidate	-0.0065 (-0.0077, -0.0053)

## Discussion

In this study, we investigated the feasibility of using prior hospitalizations to estimate AKI risk at hospital re-entry. The general objective was to extract and compress high-dimensional EHR information into a probability estimate specifically for rehospitalized patients. Performance was assessed at the patient as well as hospitalization level. Errors were also carefully analyzed to uncover gaps in predictive performance, with comprehensive analysis of diagnosis, race, gender, age, utilization, and method of AKI diagnosis. Increasing the *l*1 penalty produced a parsimonious and interpretable HPLR1 whose features correspond to a striking physiological fingerprint for AKI risk. Stability selection was performed to reinforce the results given the colinearity of features. Other interesting predictors for AKI in rehospitalized patients were found, including medications, which may enhance specification of statistical AKI models and new investigations into modifiable risk factors. While such predictive systems require extensive validation before clinical deployment, this work is a step toward creating automated AKI predictions, specifically for rehospitalized patients.

With respect to generalizeability, we stress that we do not present a “model” for AKI, but instead a mapping from input features to AKI probabilities. We reference a distinction made in Schmueli, et al. [84] between *explaining* and *predicting*. Here, we do the latter. We also reference a distinction made in Breiman, et al. [32] between *models* and *algorithms*. Here, we use the latter. An *explanatory model* would require different methods, especially with regard to model specification and dependencies in the data. We also recommend that parameters be retuned on different data for use elsewhere (“train locally”) as is commonly advised [16, 75, 85]. Thus, the systems presented here are only valid in the population from which the training data were sampled, and even there would require out-of-sample validation.

### Comparison to other AKI and EHR prediction studies

The state of the art in AKI prediction is the work of Cronin, et al. [16]. Direct comparison of performance with their models is challenging for several reasons. First, they provide predictions at a different time. We provide an at-entry risk score while Cronin et al. provides a risk score 48 hours *post-admission*. We therefore use only features from prior hospitalizations while Cronin et al. uses features from the current hospitalization (from the 48 hours between admission and prediction time) as well as prior history. Specifically, Cronin et al. used preadmission body mass index and preadmission diagnoses from -365 days to -24 hours and preadmission medications and temperatures from -90 days to -24 hours. We did not have access to body mass index or temperature, and the feature engineering required to extract other variables such as medications was labor intensive, so even a comparison of our system with their pre-admission system was not possible. Second, Cronin et al. focused specifically on hospital-acquired AKI while we focused on hospital and community acquired AKI. Third, we analyzed different cohorts. In Cronin et al., since prediction was made at 48 hours, all hospitalizations with duration less than 48 hours were excluded (roughly 1.9 million hospitalizations). In contrast, our study, in which a prediction is made at hospital re-entry, applies to any patient regardless of length of stay. We, however also excluded patients without prior hospitalizations (although we could give a prediction for these patients with no information by simply using the baseline prevalence of AKI). Therefore, in the space of all patients still present after 48 hours, the system in Cronin et al. is more general; in the space of all rehospitalized patients, our system is more general. Also, in Cronin et al., data was from Veterans Affairs hospitals and included outpatient data; we only used inpatient data from a single hospital network, not just veterans. Another similar study Kate et al. [27], analyzed strictly patients 65 years of age and older, also making comparison difficult.

Outside of AKI, the state of the art in EHR prediction has generally been achieved with RNN [86–88] or variations [89]. Here, we implemented an LSTM for sake of comparison. The LSTM implemented here was not well optimized compared to those in other studies, so it did not outperform the other systems. Nevertheless, LSTM has the clear advantage of reducing dependence on feature engineering.

### Interpretability

We do not recommend GBC, LR1, or LSTM for deployment because they are opaque. These systems make the best predictions. However, GBC, LSTM, and LR1 analyze thousands of features. In principle, a user must understand and check each of these features in order to truly explain a prediction. Otherwise, GBC or LR1 could infer that ibuprofen lowers AKI risk in an older patient with arthritis. Or, given so many candidate predictors, GBC or LR1 might rely heavily on a feature whose relationship to the response is borne of pure chance *throughout the dataset and undetectable by internal validation* [90]. Some studies [8] have recommended that tools like GBC or LR1 only be used for feature discovery, and rather that a tool similar to HPLR1 be deployed, even with some reduction in predictive performance. The user, on whom the onus falls to separate prediction from action [91], can more easily interpret HPLR1. Using fewer features especially facilitates tracing an aberrant prediction back to, for example, a data entry error. A parsimonious statistical model might even enable much needed closed-form expressions for prediction intervals (e.g., since prediction variance increases with risk). Thus, insights from this study can be used for *specification* of such a model.

We note, however, that only taking into account a few features potentially results in a system that does not adjust for variables when it should. Further, a human provider cannot analyze 1,500 features. Many of the features we analyze here are *hidden* from the EHR user. A learning algorithm that analyzes a large amount of—sometimes hidden—EHR data might thus be a useful complement. However, we cannot ignore the benefits of parsimony, so recommend that both GBC (or LR1) and HPLR1 be used *in concert* to give two separate risk scores.

### Limitations & future directions

The major limitation of this study is difficulty in validating the assumptions outlined in the methods, especially the first assumption regarding interventions that flip labels. Dependence on this assumption could be reduced by predicting sCr directly; since an intervening provider is responding to sCr, the algorithm could stay one step ahead, or by modifying the cost function to account for uncertainty in AKI status [92]. The last assumption is also difficult to validate and might lead to a system that *favors* high utilizers [93]. These difficulties arise from the fact that EHR data is not collected explicitly for predictive modeling. We also list some methodological limitations and future directions: the HP search space and the HP themselves were not conceived of independently in each fold of nested CV, but instead set manually. Bias was preferred to variance in choice of HP (and it was required that the test performance of the fold used to select HP not be optimistic relative to the other folds, a constraint much more easily fulfilled with higher bias HP). By doing so, however, the data were slightly underfit, as evidenced by the error analysis, which essentially revealed undetected patterns. This is especially apparent with respect to age. We strongly suspect that had an ideal parameter search been achieved, or had HP that allowed higher variance been permitted, the GBC could have detected most of these patterns, and the error analysis would not have revealed such biases. This, however, might have increased risk of overfitting. At least bias is possible to detect (as we have done) whereas overfitting can be elusive. Given the high number of predictors (especially relative to the cases), GBC and LR1 are likely overfit (not in the traditional sense, which can be detected via internal validation) but to peculiarities of the entire dataset, impossible to determine with internal validation alone. We however, via domain-expertise-guided evaluation of features, consider this study to still contain insights of value to this prediction problem and cohort.

Administrative codes are problematic predictors. Although codes may be embedded or otherwise optimized as features [38, 94], such approaches are not straightforward to implement in a pipeline. Also, past AKI is a good predictor of future AKI. Numerous reports suggest that codes have low sensitivity for AKI. Therefore, using code-based AKI as a predictor is not ideal. AKI as a target was supplemented with sCr; AKI as a predictor was not supplemented with sCr, however, as this would necessitate extensive preprocessing of sCr trajectories in real time if deployed (time series models could take care of this for free, however). For missing data imputation, more careful classification of missingness and more sophisticated methods such as matrix completion should be explored in the future. For laboratory values, Gaussian processes have also shown good performance [88].

Codes are also problematic as targets. Although sCr-based diagnoses were used to supplement codes, we noted high discrepancy between the two. Visual inspection suggests that sCr for hospitalizations diagnosed by code but not sCr usually began above normal and then decreased during the hospital stay, suggesting that an outpatient reading, or even a high initial measurement, prompted code assignment. Without these cases, our findings align with previous reports that codes are specific but not sensitive for AKI. It was also apparent that errors were slightly higher in the cases diagnosed by sCr but not by code. Another difficulty with diagnosis codes as labels is that they are often assigned at the end of the hospitalization and therefore not time stamped. It is impossible therefore to know when the AKI occurred during the hospitalization (i.e., we do not distinguish between hospital- and community-acquired AKI). On a similar note, because the majority of AKI codes were of “unspecified” severity, it was not possible to distinguish severities of AKI. This issue could be alleviated by predicting sCr directly in future work. Also relevant but not assessed is the performance of the systems as a function of time as analyzed in [15]. For example, certain medications might wane in popularity or diseases might be seasonal. We hope to asssess this in the future and analyze the effect of online training.

## Conclusion

This study gives insight into the EHR-based AKI prediction problem in rehospitalized patients. Our objective was to investigate the feasibility of predicting AKI in this cohort as well as to analyze some interesting predictors. We trained several learning algorithms and perform an in-depth error analysis, looking for specific patient groups for which predictions might be poor. We also revealed novel predictors that could be used for specification of a statistical model. We further focused on pharmaceutical predictors that may be worth further exploration as modifiable risk factors. We consider this work a step towards an automated, locally-trained tool that leverages sometimes hidden, longitudinal EHR data to estimate AKI risk in rehospitalized patients without manual ordering of tests, data collection, or data entry. Such an estimate could provide a prior probability at the time of hospital re-entry to be used by an admitting provider or another predictive algorithm.

## Supporting information

S1 File Algorithm specifications. (PDF)

S1 Fig Metric distributions. Metric distributions over the 250 inner folds are shown.
(TIF)

S2 FigError distributions by diagnosis method.We show the distributions of error, $|\hat{y}-y|$ where *y* is a binary label and $\hat{y}$ is the probability estimate, by diagnosis method. “∨” corresponds to cases where diagnosis was made either by code or sCr; “∧” corresponds to cases in which diagnosis was made by both code and sCr; “-” indicates a set difference. Histograms have 1000 bins.(TIF)

S3 Fig LR1 evaluation. ROC, Calibration, and PR curves for 50 iterations of 5-fold CV for LR1.
(TIF)

S4 Fig ALR1 evaluation. ROC, Calibration, and PR curves for 50 iterations of 5-fold CV for the Anscombe LR1.
(TIF)

S5 Fig RLR1 evaluation. ROC, Calibration, and PR curves for 50 iterations of 5-fold CV for the randomized LR1.
(TIF)

S6 Fig HPLR1 evaluation. ROC, Calibration, and PR curves for 50 iterations of 5-fold CV for the highly penalized LR1.
(TIF)

S7 Fig RHPLR1 evaluation. ROC, Calibration, and PR curves for 50 iterations of 5-fold CV for the randomized highly penalized LR1.
(TIF)

S8 Fig WGBC evaluation. ROC, Calibration, and PR curves for 50 iterations of 5-fold CV for weighted GBC.
(TIF)

S9 FigWGBC calibration.Observed hospitalization-level risk is plotted against predicted risk (top) and patient-level mean observed risk against mean predicted risk (bottom). In the scatter plots, alpha level is 0.05 and the red calibration curve corresponds to all hospitalizations or to patients who had either mean risk over hospitalizations of 1 or 0. The calibration curves are computed according to the macro-averaged predicted output per hospitalization or patient over the 50 iterations of 5 fold CV (over 250 total folds). Ideal calibration is the dotted black diagonal. *P*_*O*_ = observed risk per hospitalization, *P*_*P*_ = predicted risk per hospitalization, $\bar{P_{O}}$ = mean observed risk over hospitalizations, $\bar{P_{P}}$ = mean predicted risk over hospitalizations.(TIF)

S10 Fig WGBC utilization. The mean and STD absolute error is shown as a function of the number of hospitalizations. Patients were binned based on the number of hospitalizations in the dataset and then, over bins, the mean error and STD of the predictions were computed. Stratification by outcome is performed since it was earlier established that the hospitalization:patient ratio is higher in cases than in controls.
(TIF)

S11 Fig WLR1 evaluation. ROC, Calibration, and PR curves for 50 iterations of 5-fold CV for weighted LR1.
(TIF)

S12 Fig WHPLR1 evaluation. ROC, Calibration, and PR curves for 50 iterations of 5-fold CV for weighted HPLR1.
(TIF)

S13 Fig SGBC evaluation. ROC, Calibration, and PR curves for 50 iterations of 5-fold CV for sampled GBC.
(TIF)

S14 Fig SLR1 evaluation. ROC, Calibration, and PR curves for 50 iterations of 5-fold CV for sampled LR1.
(TIF)

S15 Fig SHPLR1 evaluation. ROC, Calibration, and PR curves for 50 iterations of 5-fold CV for sampled HPLR1.
(TIF)

S16 Fig RGBC evaluation. ROC, Calibration, and PR curves for 50 iterations of 5-fold CV for the RGBC using features from only the most recent hospitalization rather than all available prior hospitalizations.
(TIF)

S17 Fig MGBC evaluation. ROC, Calibration, and PR curves for 50 iterations of 5-fold CV for the MGBC trained only on medications.
(TIF)

S18 Fig MLR1 evaluation. ROC, Calibration, and PR curves for 50 iterations of 5-fold CV for the MLR1 trained only on medications.
(TIF)

S19 Fig CLR evaluation. ROC, Calibration, and PR curves for 50 iterations of 5-fold CV for clinical LR.
(TIF)

S20 Fig LSTM evaluation. ROC, Calibration, and PR curves for 50 iterations of 5-fold CV for LSTM.
(TIF)

S21 Fig NGBC evaluation. ROC, Calibration, and PR curves for 50 iterations of 5-fold CV for GBC trained on permuted response. The identity for the calibration curve was hidden and the alpha value set to 1 for better visualization.
(TIF)

S22 Fig NGBC utilization. The mean and STD absolute error is shown as a function of the number of hospitalizations. Patients were binned based on the number of hospitalizations in the dataset and then, over bins, the mean error and STD of the predictions were computed. Stratification by outcome is performed since it was earlier established that the hospitalization:patient ratio is higher in cases than in controls.
(TIF)

S23 Fig NGBC prediction variance. The mean and standard deviation of predicted probabilities are plotted over iterations (per hospitalization). Alpha = 0.01 for all plots.
(TIF)

S24 Fig GBC error by age. Alpha = 0.01. The top (in red, lighter) are the cases and the bottom (in blue, darker) are the controls.
(TIF)

S25 Fig HPLR1 coefficient perturbation by utilization. Influence over coefficients of HPLR1 vs. utilization is shown for each patient with two or more hospitalizations. Distance between coefficient vectors was computed using the l1 norm.
(TIF)

S1 Table Regression results for error analysis. Shown is the choice of HP Alpha and the train, validation, and test mean squared error (MSE) of the regression from the fold in which Alpha was chosen.
(PDF)

S2 Table Feature importances/coefficients for RLR1. For laboratory results, the first function is *G*, aggregation over hospitalizations, and the second is *F*, aggregation within a hospitalization; e.g., “mean max sCr” is the mean over hospitalizations of the maximum sCr of each hospitalization.
(PDF)

S3 Table Coefficients of RHPLR1. For laboratory results, the first function is *G*, aggregation over hospitalizations, and the second is *F*, aggregation within a hospitalization; e.g., “mean max sCr” is the mean over hospitalizations of the maximum sCr of each hospitalization.
(PDF)
