# Supplementary material for: Predicting acute kidney injury at hospital re-entry using high-dimensional electronic health record data
Source: PLoS One. 2018 Nov 20;13(11):e0204920. doi: 10.1371/journal.pone.0204920 (PMC6245516; doi:10.1371/journal.pone.0204920)
Supplement: S1 File — (PDF) [file pone.0204920.s001.pdf]

GBC: loss=deviance, learning rate=0.1, n estimators=100, subsample=1.0, criterion=friedman mse, min samples split=150, min samples leaf=100, min weight fraction leaf=0.0, max depth=2, min impurity split=1e-07, init=None, class weight=balanced random state=random state, max features=None, verbose=0, max leaf nodes=None, warm start=False, presort=auto

LR1: penalty=l1, dual=False, tol=0.0001, C=2e-3 (LR1) or 2e-4 (HPLR1), fit intercept=True, intercept scaling=1, class weight=balanced, random state=random state, solver=liblinear (uses coordinate descent), max iter=100, multi class=ovr, verbose=0, warm start=False, n jobs=5

Lasso: alpha=for diagnoses cases 0.015, for controls 10e-5; for other stratifiers, 0, fit intercept=True, normalize=False, precompute=False, copy X=True, max iter=1000, tol=0.0001, warm start=False, positive=False, selection=cyclic

Randomized Logistic Regression: C=0.5 for RLR1 and 0.2 for RHPLR1, sample fraction=0.74, n resampling=50 pipe to lasso with C=1, class weights=balanced

LSTM: optimizer=adam, epochs=4, batch size=500, layer 1 hidden units=30, layer 2 hidden units=20, dropout=random(0.25, 0.50, 0.75), iterations random search=3, score random search=log loss
