# Supplementary figures and images for: Predicting acute kidney injury at hospital re-entry using high-dimensional electronic health record data
Source: PLoS One. 2018 Nov 20;13(11):e0204920. doi: 10.1371/journal.pone.0204920 (PMC6245516; doi:10.1371/journal.pone.0204920)

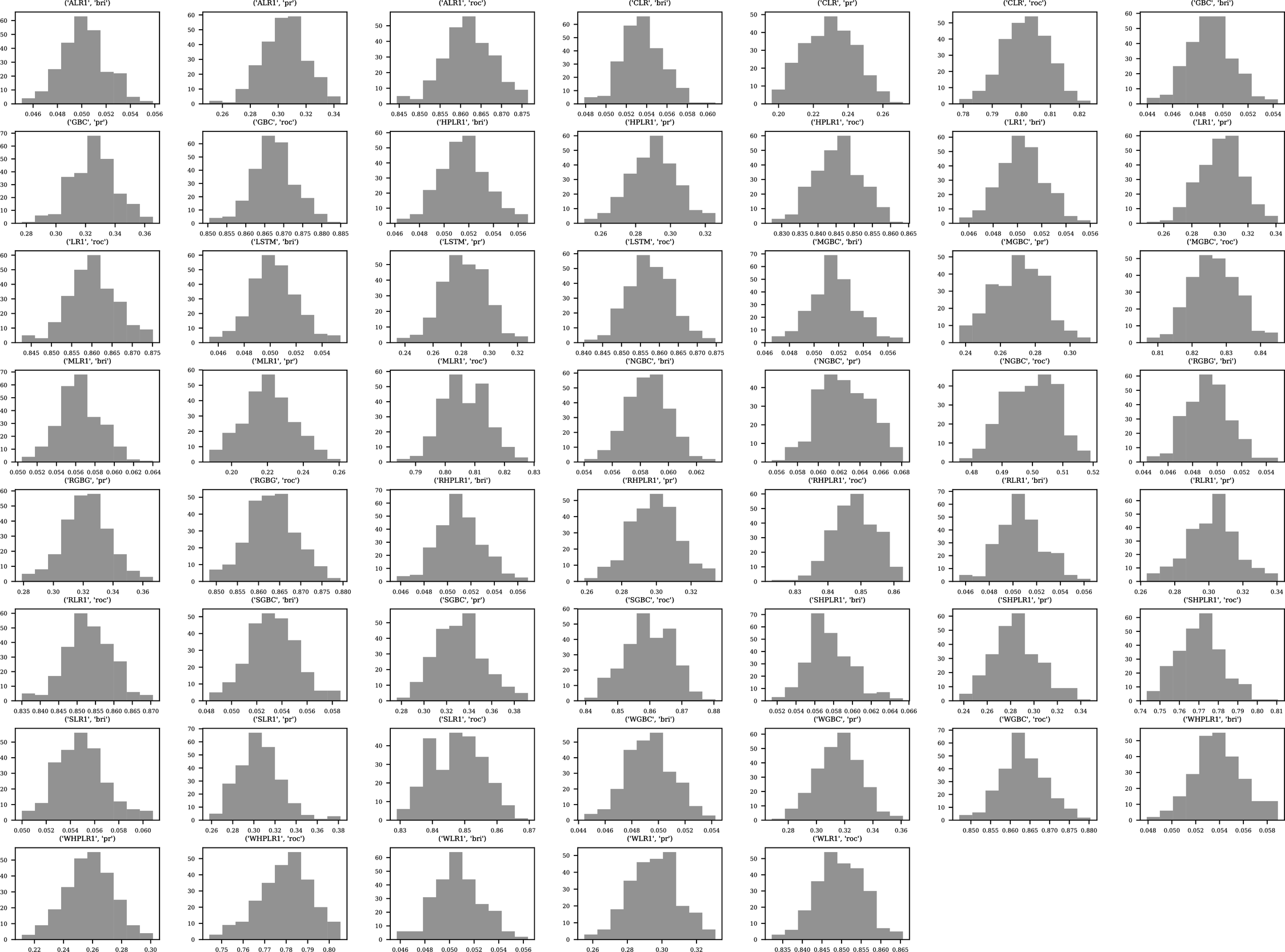

Supplement: S1 Fig — Metric distributions over the 250 inner folds are shown. (TIF) [file pone.0204920.s002.tif]

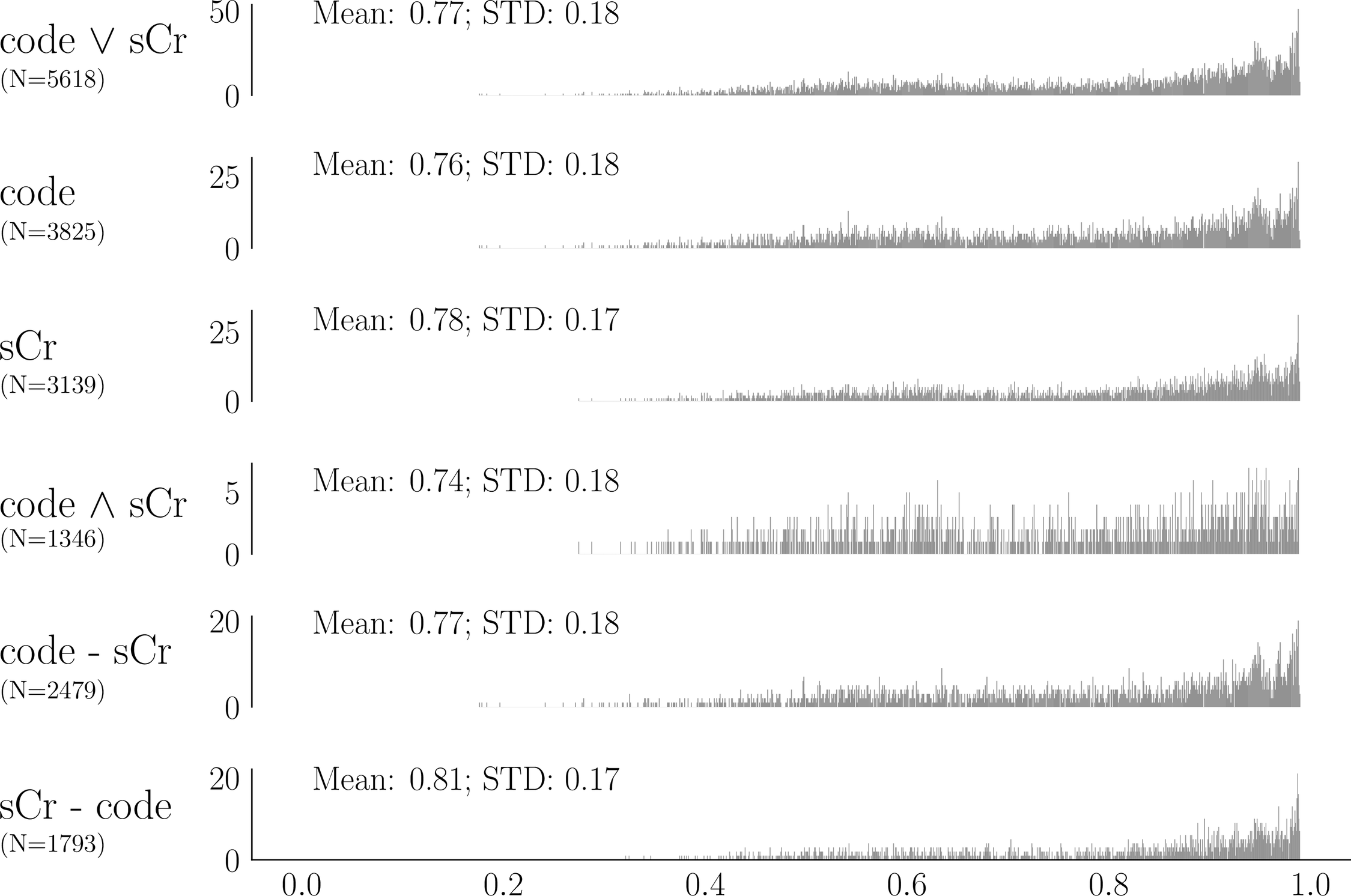

Supplement: S2 Fig — We show the distributions of error, |y^-y| where y is a binary label and y^ is the probability estimate, by diagnosis method. “∨” corresponds to cases where diagnosis was made either by code or sCr; “∧” corresponds to cases in which diagnosis was made by both code and sCr; “-” indicates a set difference. Histograms have 1000 bins. (TIF) [file pone.0204920.s003.tif]

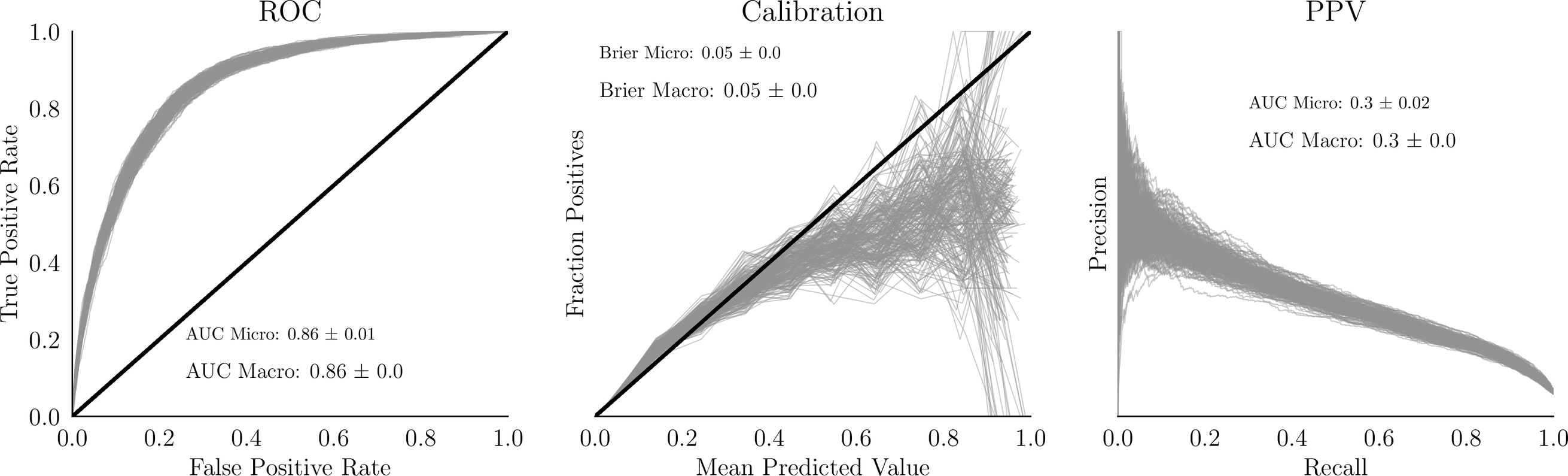

Supplement: S3 Fig — ROC, Calibration, and PR curves for 50 iterations of 5-fold CV for LR1. (TIF) [file pone.0204920.s004.tif]

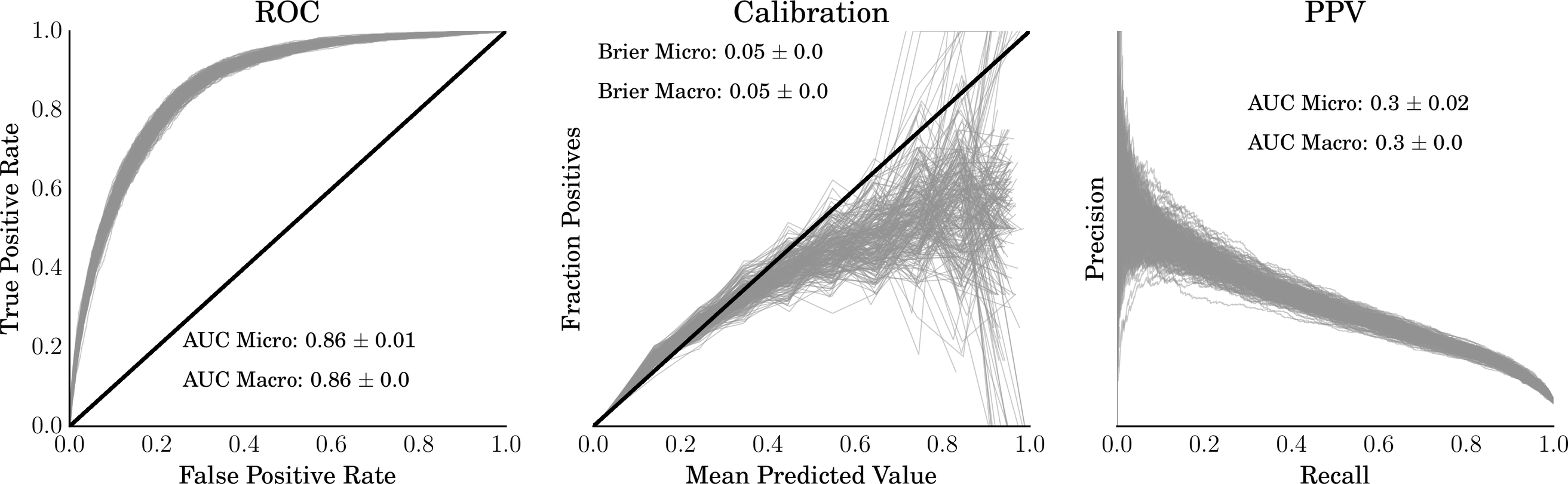

Supplement: S4 Fig — ROC, Calibration, and PR curves for 50 iterations of 5-fold CV for the Anscombe LR1. (TIF) [file pone.0204920.s005.tif]

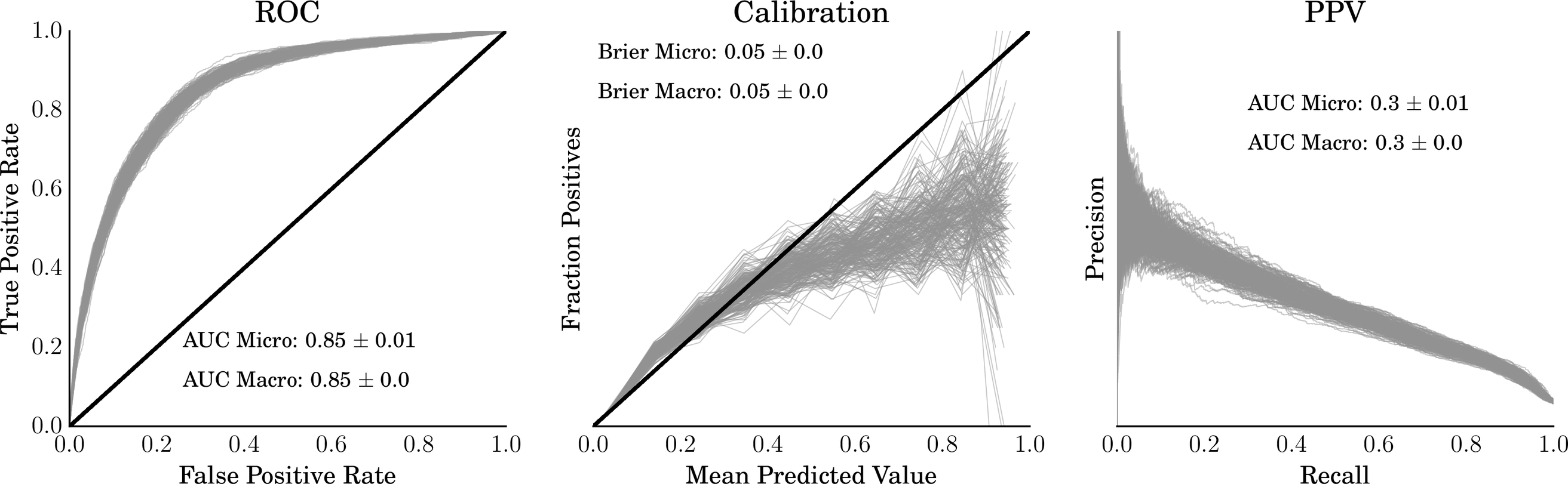

Supplement: S5 Fig — ROC, Calibration, and PR curves for 50 iterations of 5-fold CV for the randomized LR1. (TIF) [file pone.0204920.s006.tif]

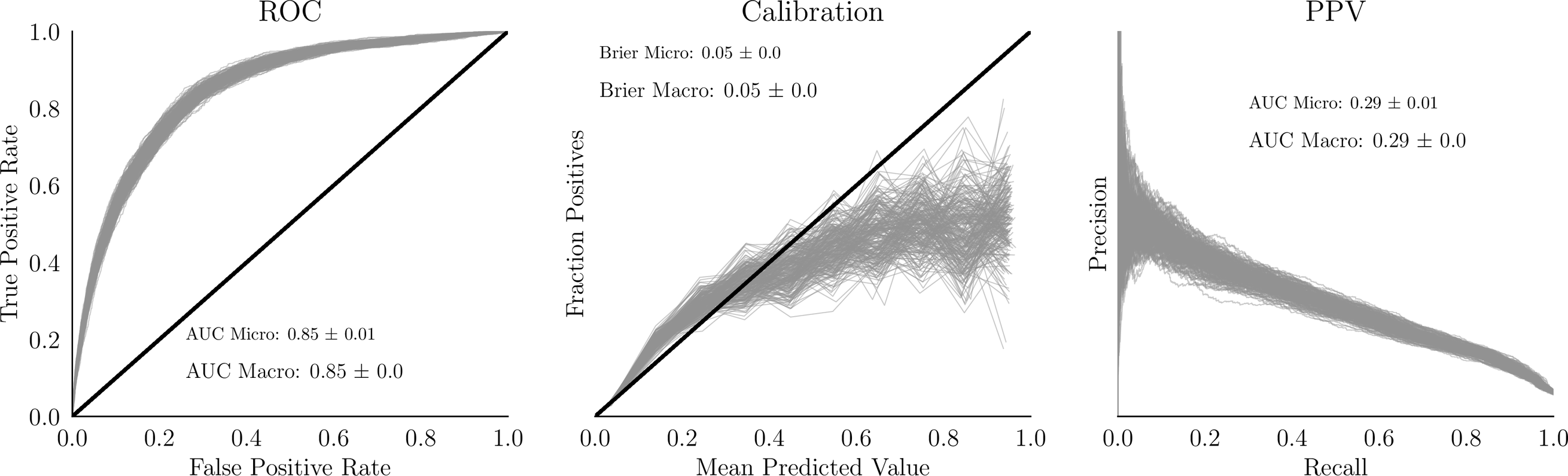

Supplement: S6 Fig — ROC, Calibration, and PR curves for 50 iterations of 5-fold CV for the highly penalized LR1. (TIF) [file pone.0204920.s007.tif]

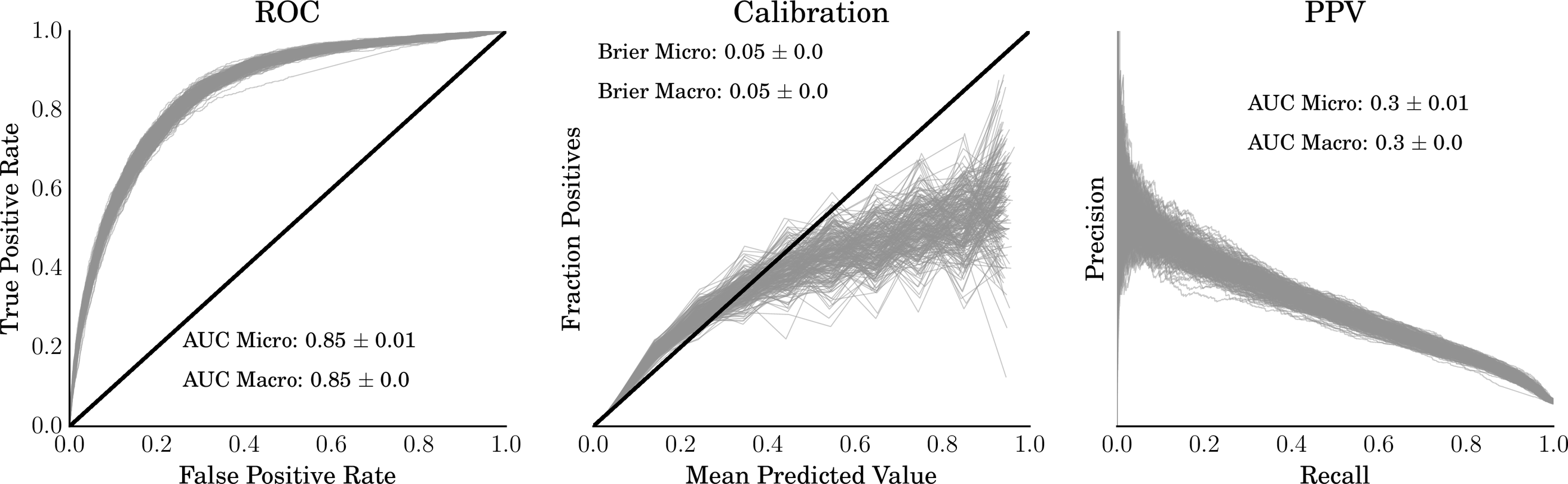

Supplement: S7 Fig — ROC, Calibration, and PR curves for 50 iterations of 5-fold CV for the randomized highly penalized LR1. (TIF) [file pone.0204920.s008.tif]

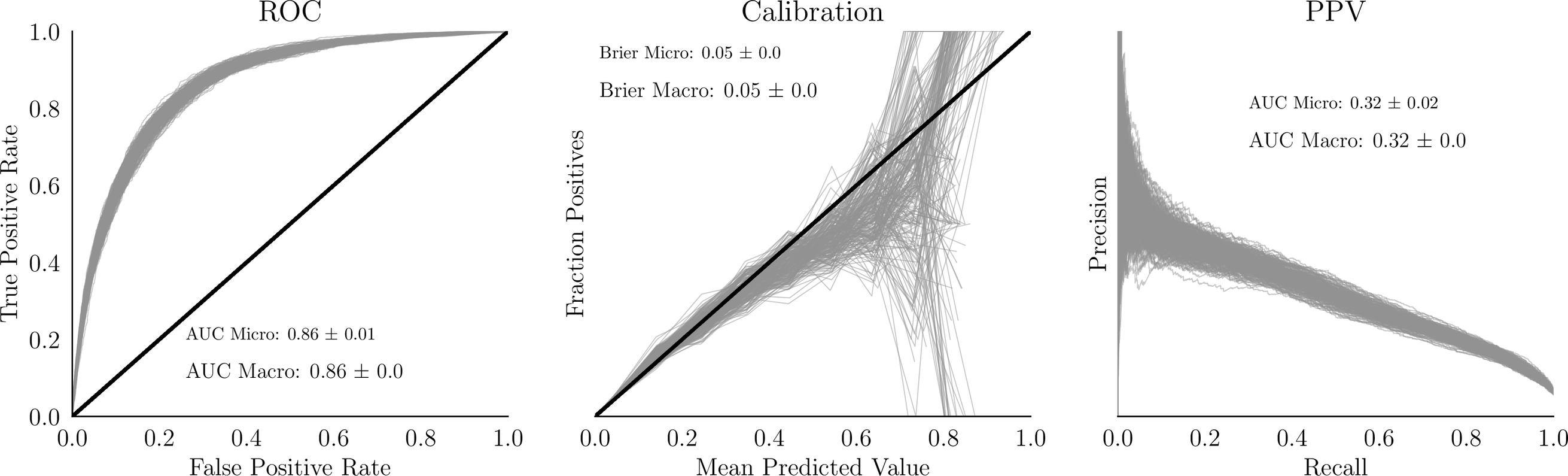

Supplement: S8 Fig — ROC, Calibration, and PR curves for 50 iterations of 5-fold CV for weighted GBC. (TIF) [file pone.0204920.s009.tif]

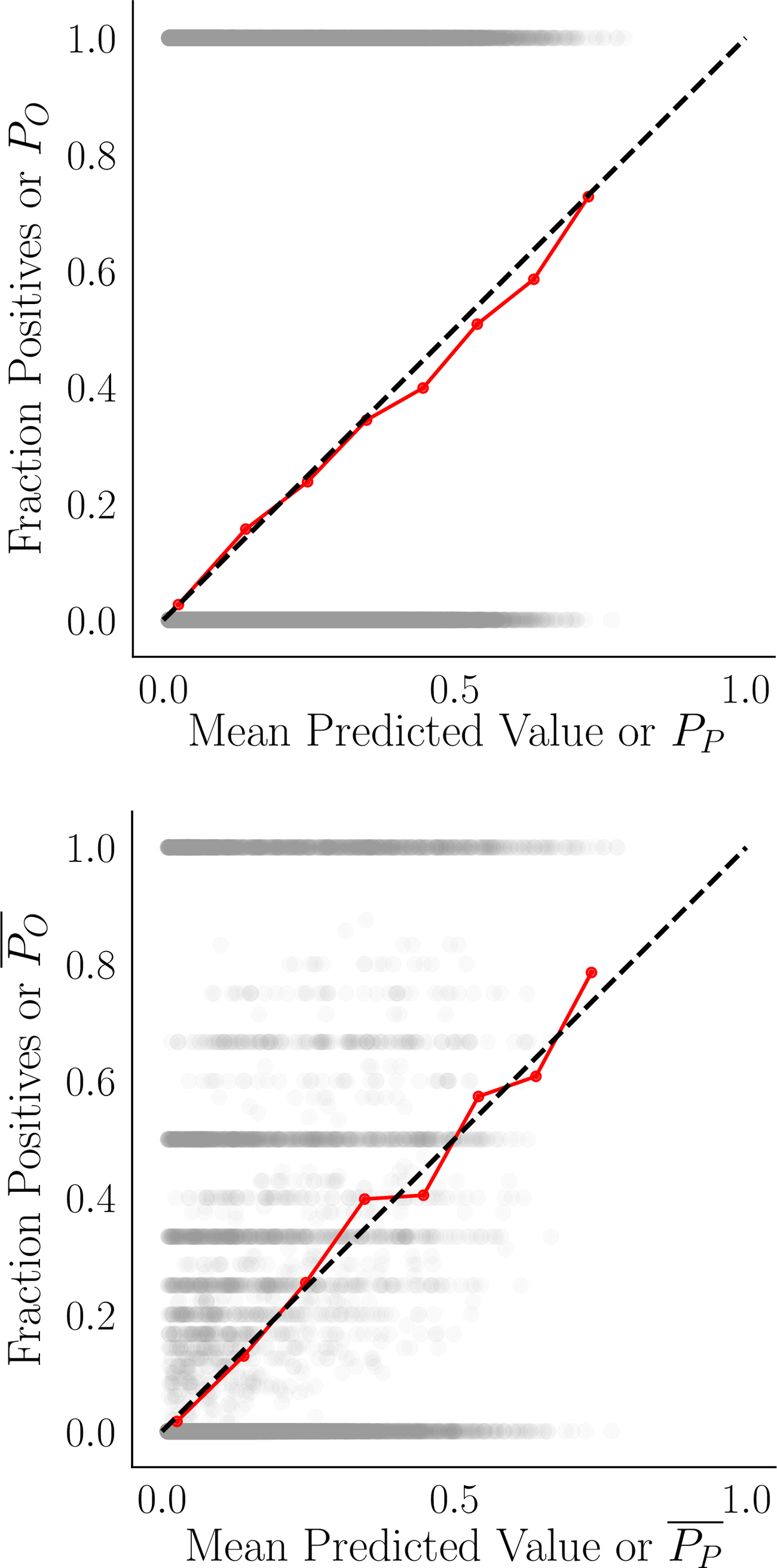

Supplement: S9 Fig — Observed hospitalization-level risk is plotted against predicted risk (top) and patient-level mean observed risk against mean predicted risk (bottom). In the scatter plots, alpha level is 0.05 and the red calibration curve corresponds to all hospitalizations or to patients who had either mean risk over hospitalizations of 1 or 0. The calibration curves are computed according to the macro-averaged predicted output per hospitalization or patient over the 50 iterations of 5 fold CV (over 250 total folds). Ideal calibration is the dotted black diagonal. PO = observed risk per hospitalization, PP = predicted risk per hospitalization, PO¯ = mean observed risk over hospitalizations, PP¯ = mean predicted risk over hospitalizations. (TIF) [file pone.0204920.s010.tif]

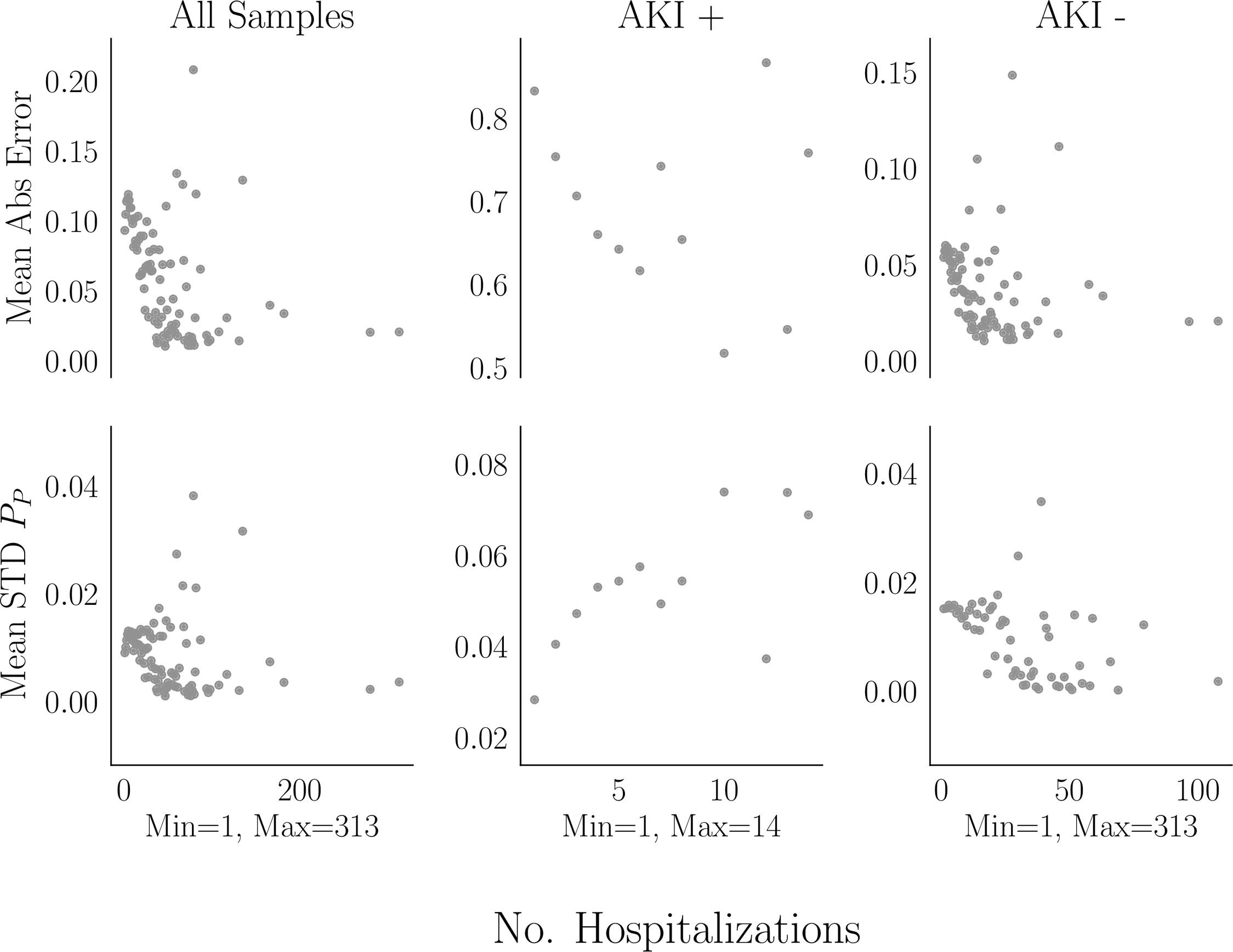

Supplement: S10 Fig — The mean and STD absolute error is shown as a function of the number of hospitalizations. Patients were binned based on the number of hospitalizations in the dataset and then, over bins, the mean error and STD of the predictions were computed. Stratification by outcome is performed since it was earlier established that the hospitalization:patient ratio is higher in cases than in controls. (TIF) [file pone.0204920.s011.tif]

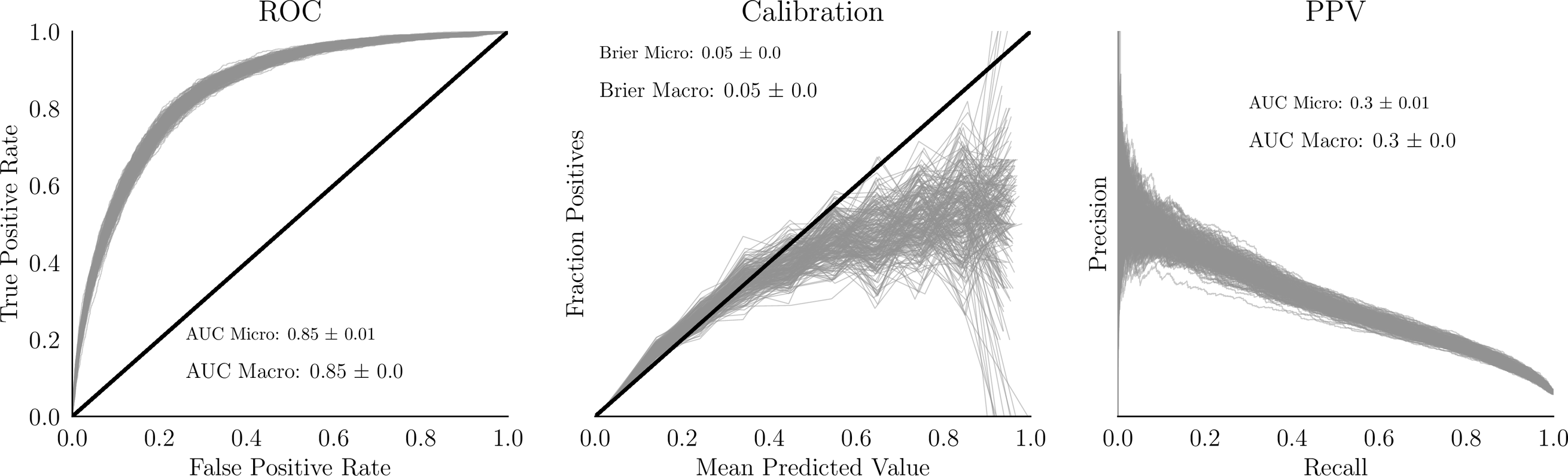

Supplement: S11 Fig — ROC, Calibration, and PR curves for 50 iterations of 5-fold CV for weighted LR1. (TIF) [file pone.0204920.s012.tif]

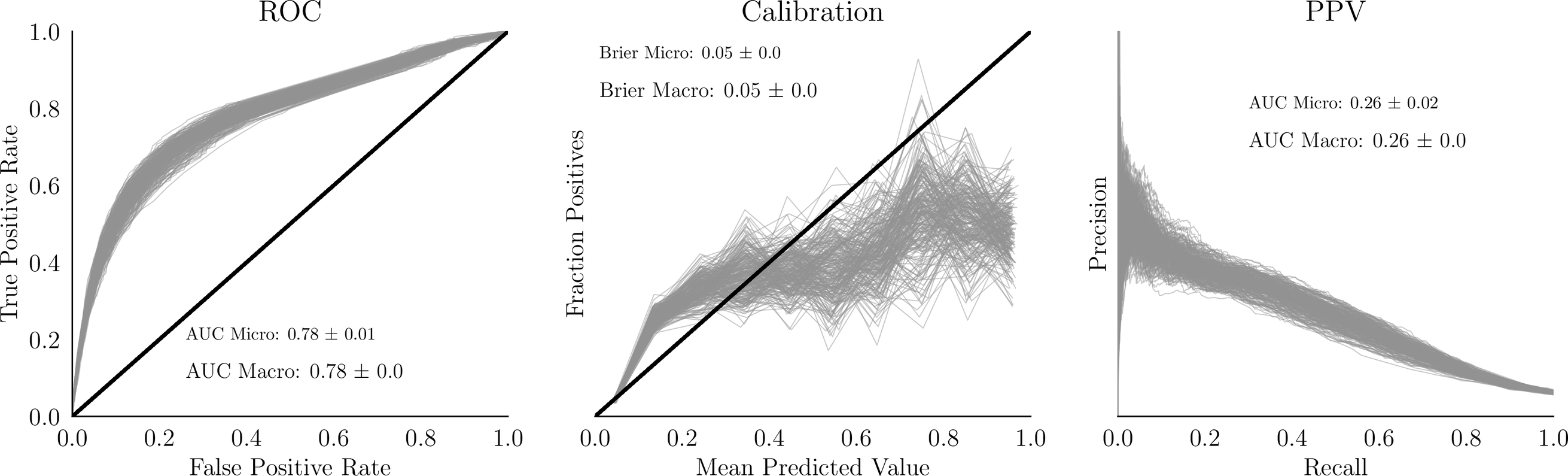

Supplement: S12 Fig — ROC, Calibration, and PR curves for 50 iterations of 5-fold CV for weighted HPLR1. (TIF) [file pone.0204920.s013.tif]

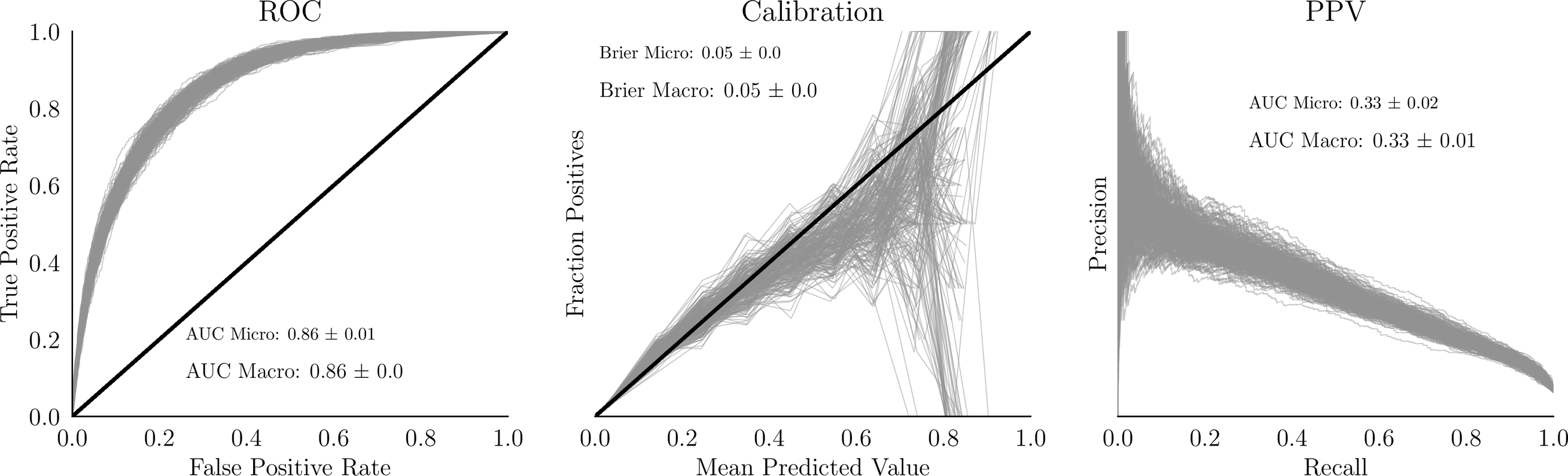

Supplement: S13 Fig — ROC, Calibration, and PR curves for 50 iterations of 5-fold CV for sampled GBC. (TIF) [file pone.0204920.s014.tif]

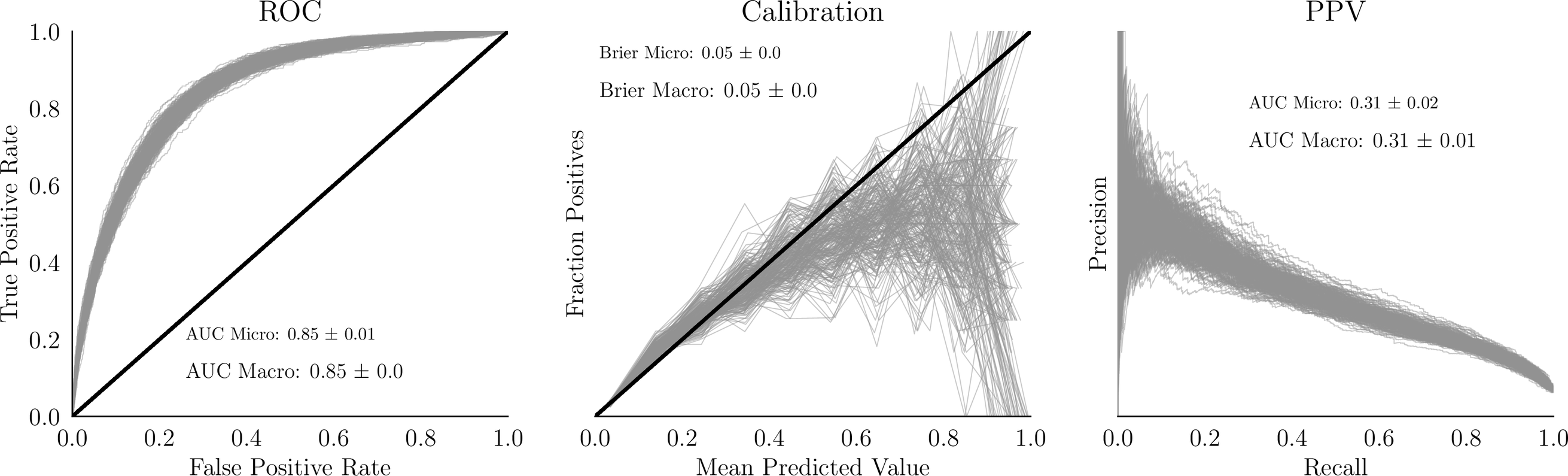

Supplement: S14 Fig — ROC, Calibration, and PR curves for 50 iterations of 5-fold CV for sampled LR1. (TIF) [file pone.0204920.s015.tif]

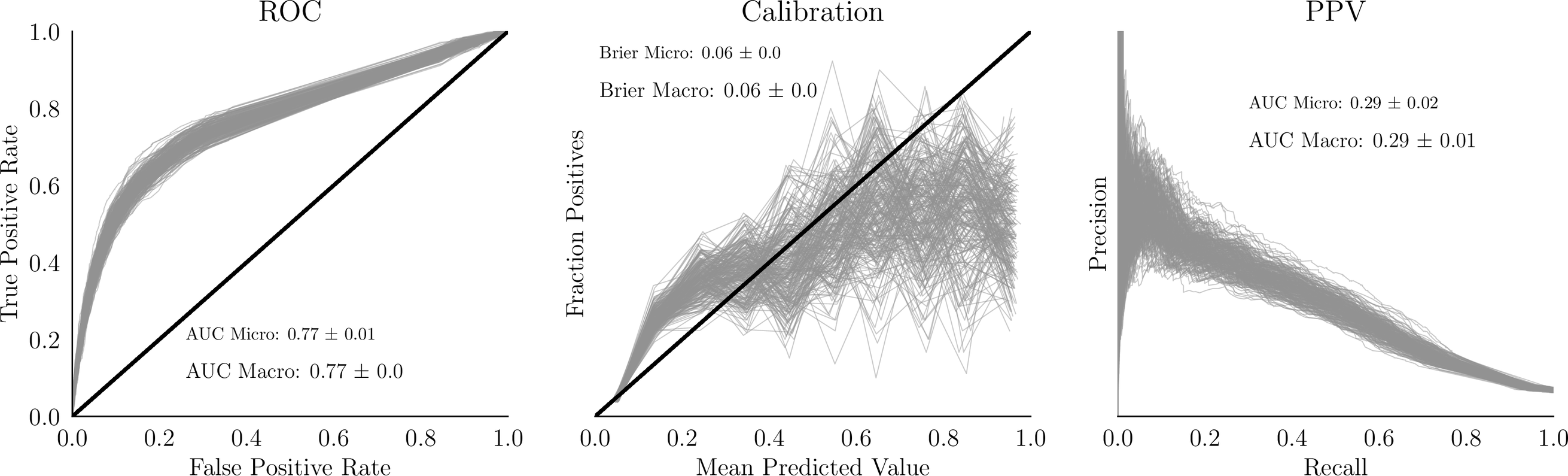

Supplement: S15 Fig — ROC, Calibration, and PR curves for 50 iterations of 5-fold CV for sampled HPLR1. (TIF) [file pone.0204920.s016.tif]

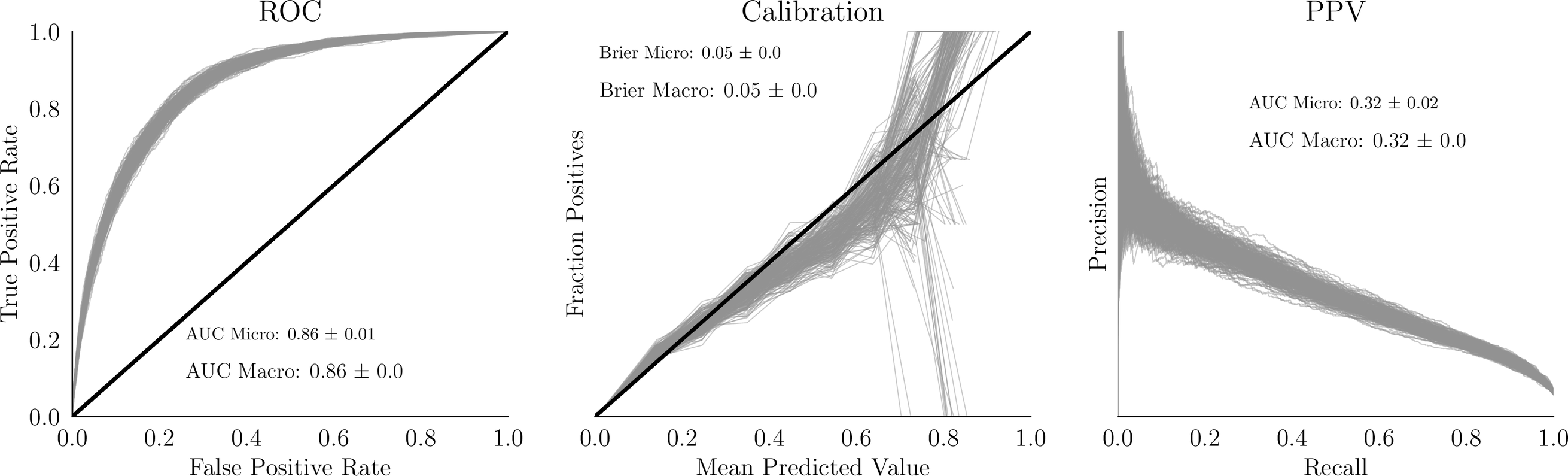

Supplement: S16 Fig — ROC, Calibration, and PR curves for 50 iterations of 5-fold CV for the RGBC using features from only the most recent hospitalization rather than all available prior hospitalizations. (TIF) [file pone.0204920.s017.tif]

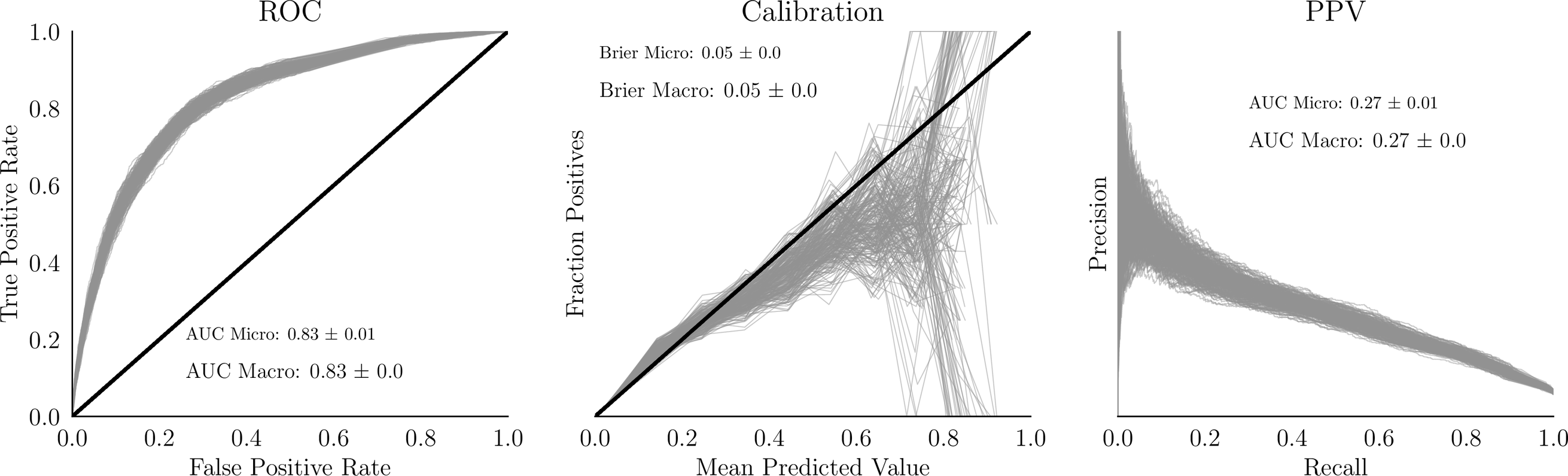

Supplement: S17 Fig — ROC, Calibration, and PR curves for 50 iterations of 5-fold CV for the MGBC trained only on medications. (TIF) [file pone.0204920.s018.tif]

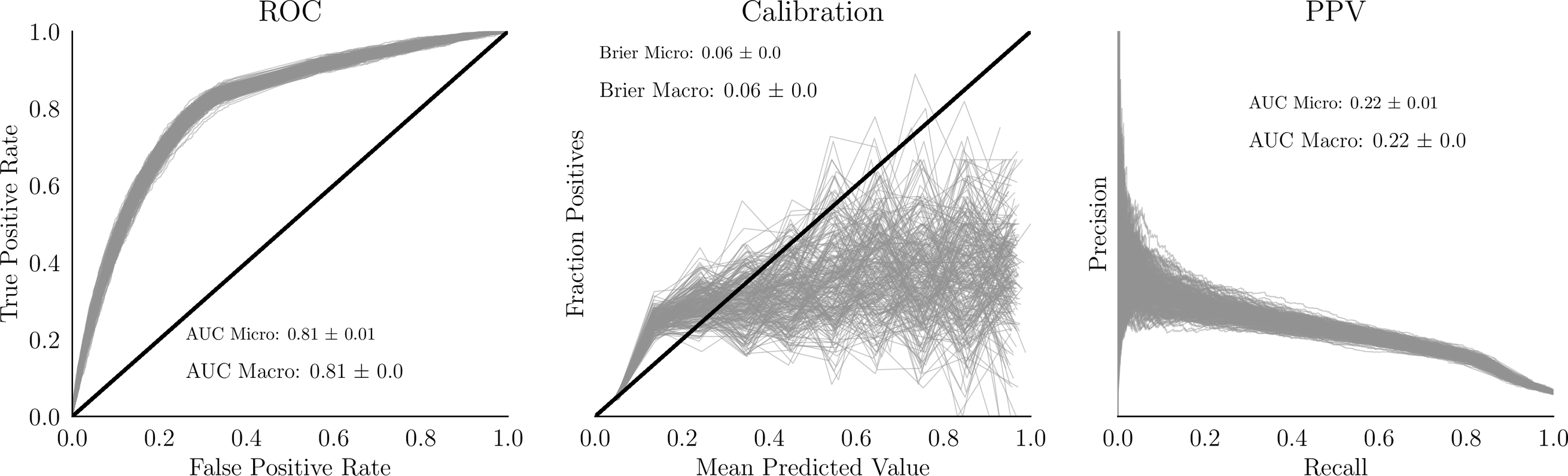

Supplement: S18 Fig — ROC, Calibration, and PR curves for 50 iterations of 5-fold CV for the MLR1 trained only on medications. (TIF) [file pone.0204920.s019.tif]

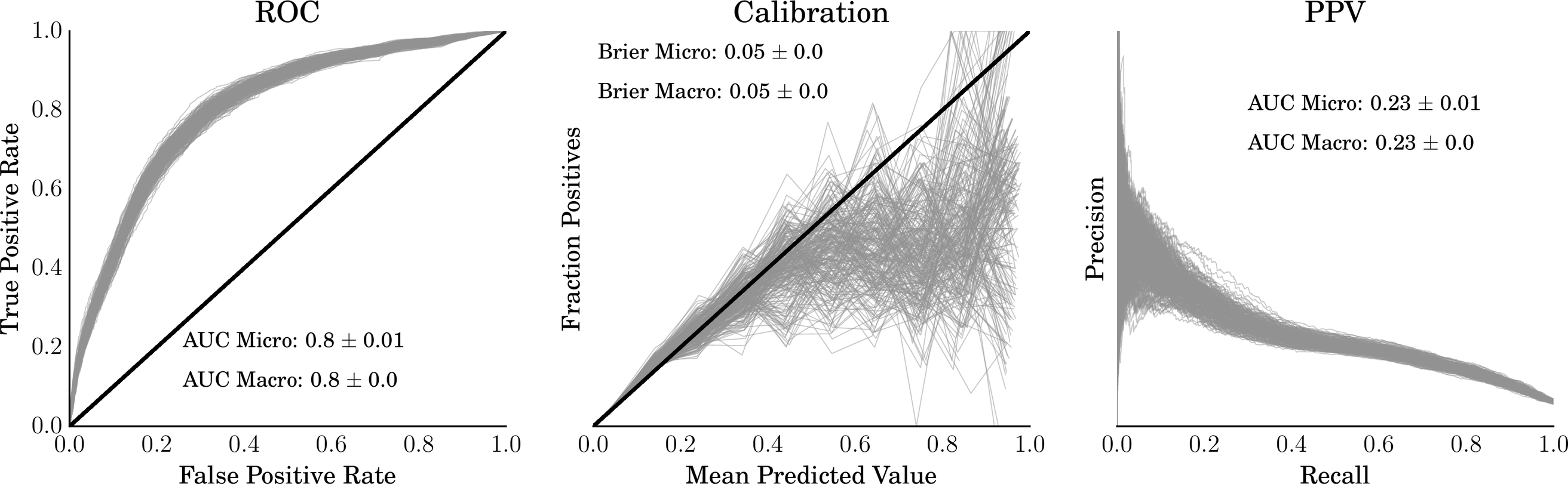

Supplement: S19 Fig — ROC, Calibration, and PR curves for 50 iterations of 5-fold CV for clinical LR. (TIF) [file pone.0204920.s020.tif]

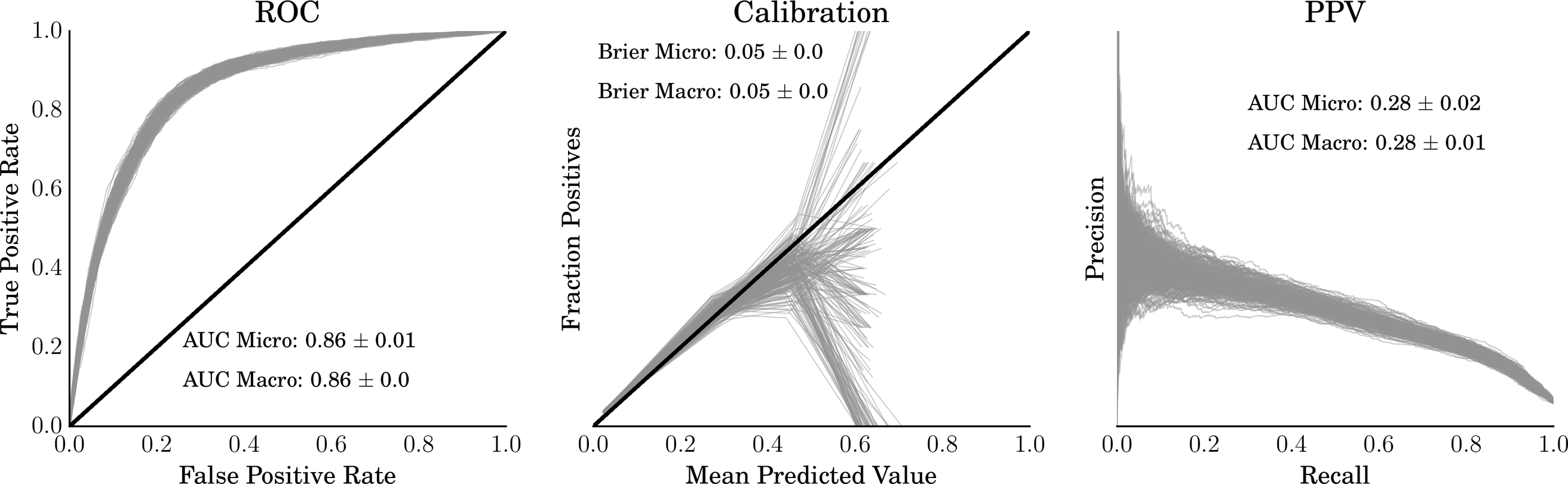

Supplement: S20 Fig — ROC, Calibration, and PR curves for 50 iterations of 5-fold CV for LSTM. (TIF) [file pone.0204920.s021.tif]

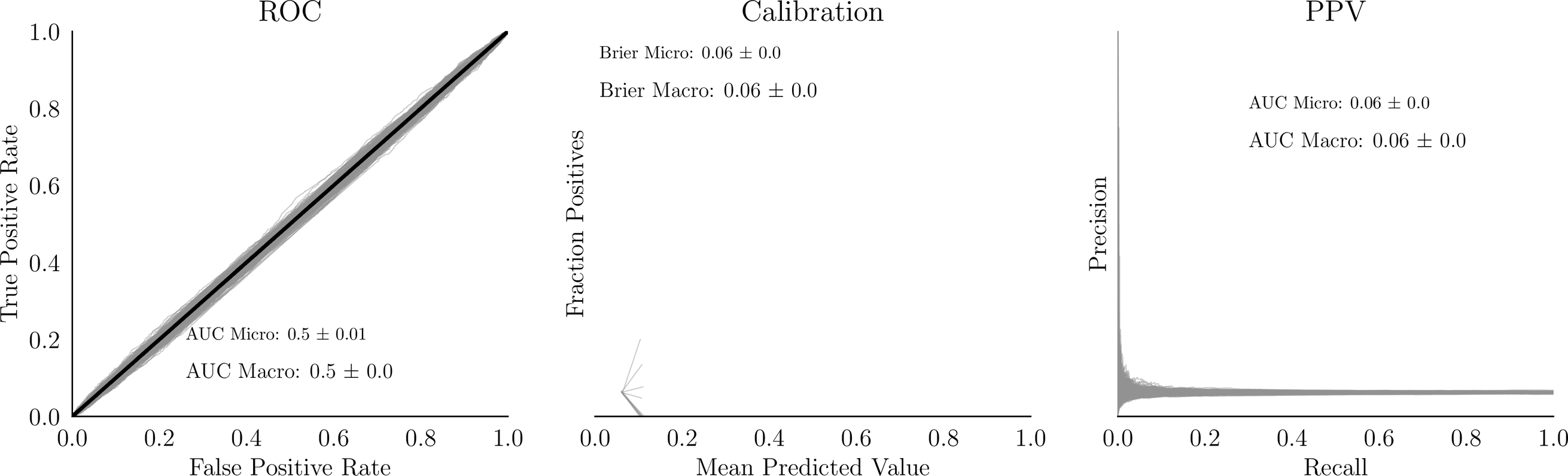

Supplement: S21 Fig — ROC, Calibration, and PR curves for 50 iterations of 5-fold CV for GBC trained on permuted response. The identity for the calibration curve was hidden and the alpha value set to 1 for better visualization. (TIF) [file pone.0204920.s022.tif]

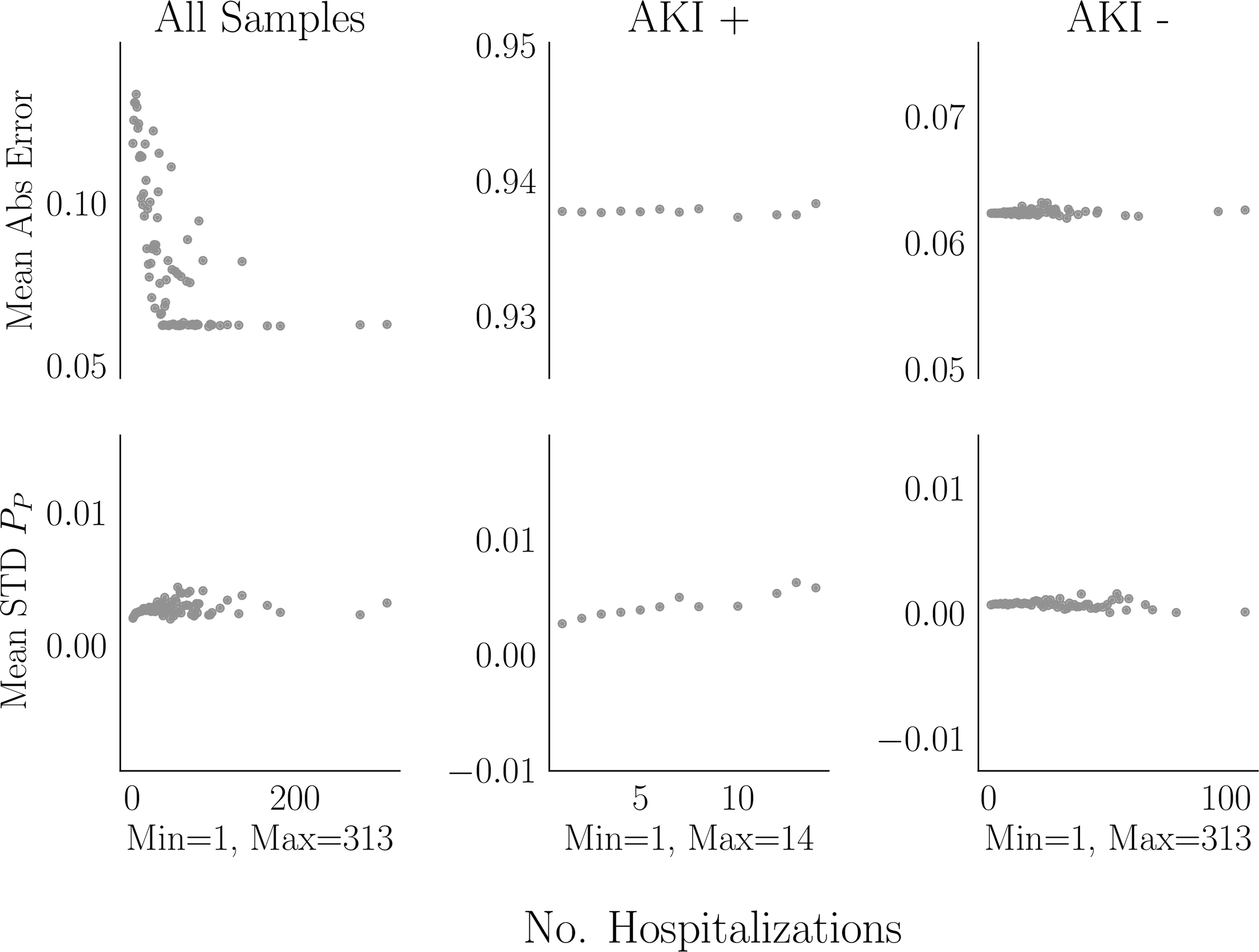

Supplement: S22 Fig — The mean and STD absolute error is shown as a function of the number of hospitalizations. Patients were binned based on the number of hospitalizations in the dataset and then, over bins, the mean error and STD of the predictions were computed. Stratification by outcome is performed since it was earlier established that the hospitalization:patient ratio is higher in cases than in controls. (TIF) [file pone.0204920.s023.tif]

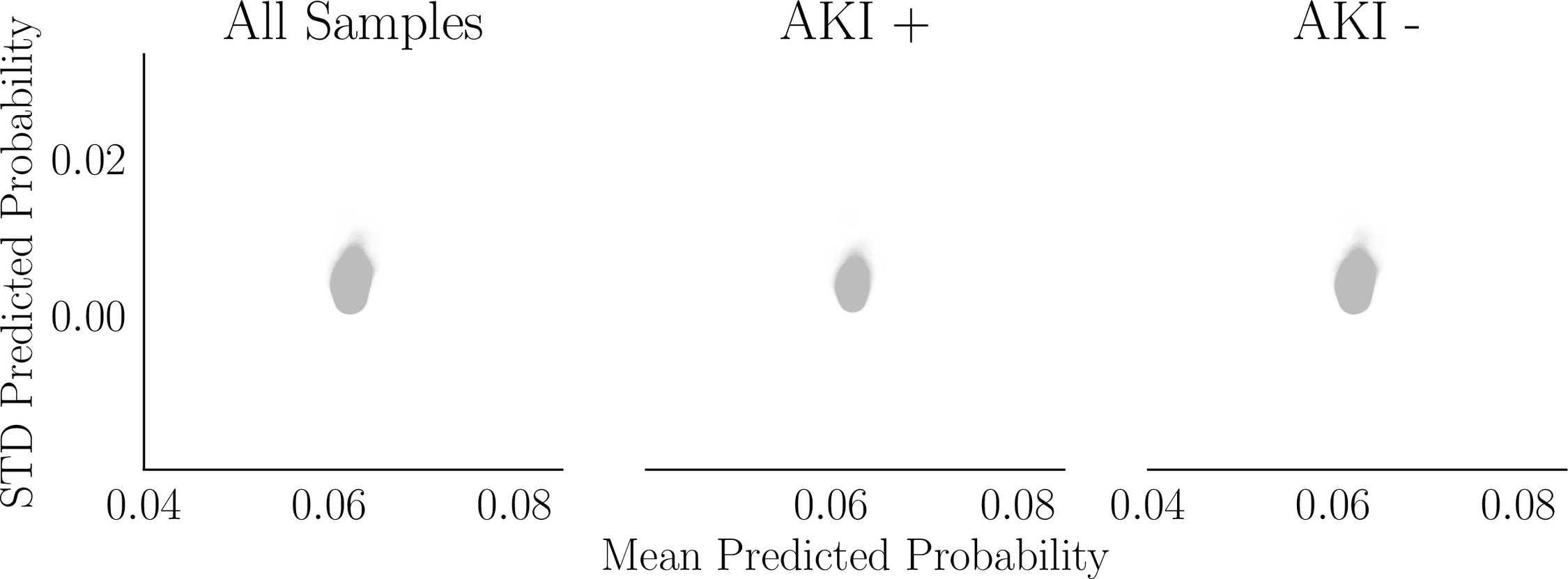

Supplement: S23 Fig — The mean and standard deviation of predicted probabilities are plotted over iterations (per hospitalization). Alpha = 0.01 for all plots. (TIF) [file pone.0204920.s024.tif]

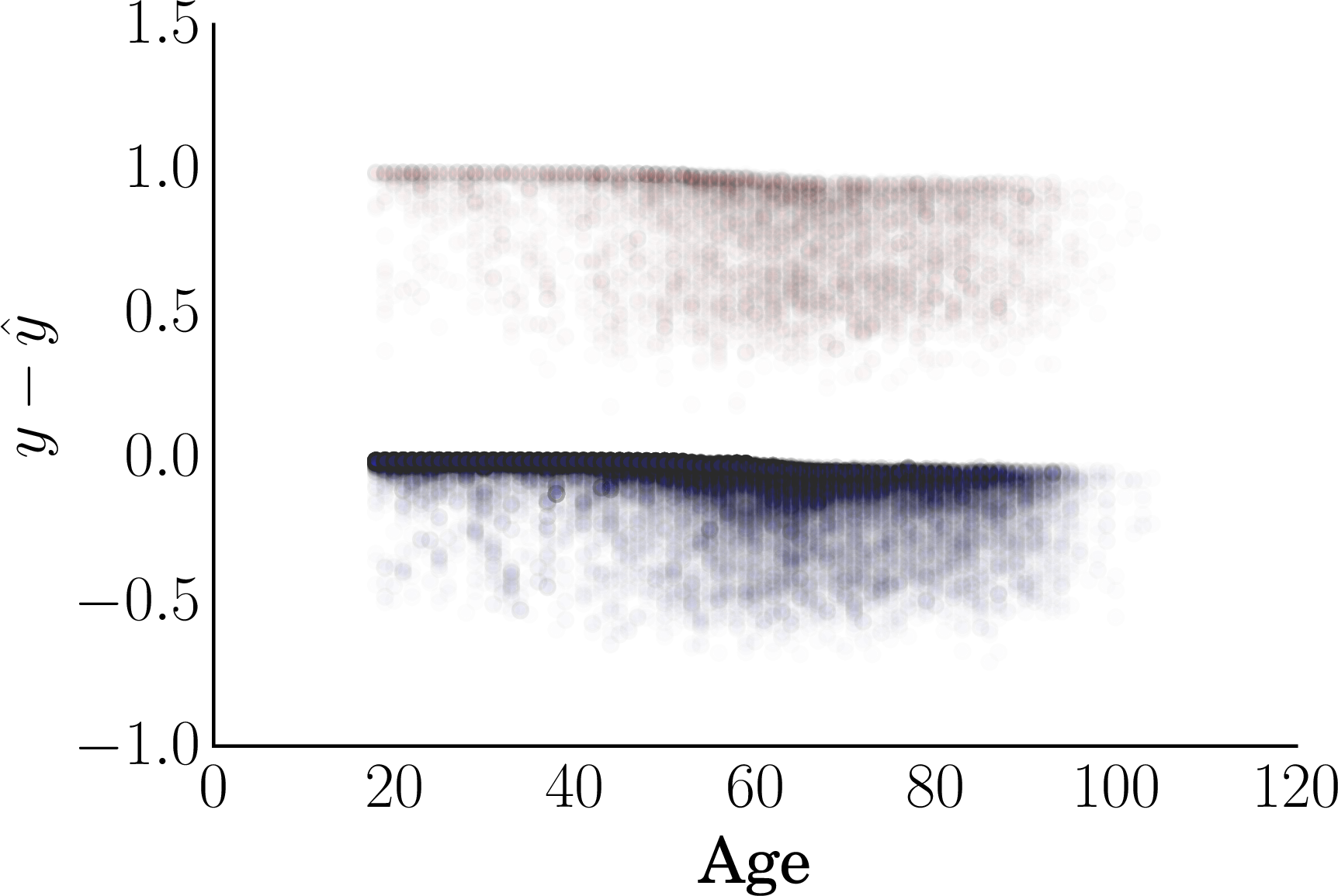

Supplement: S24 Fig — Alpha = 0.01. The top (in red, lighter) are the cases and the bottom (in blue, darker) are the controls. (TIF) [file pone.0204920.s025.tif]

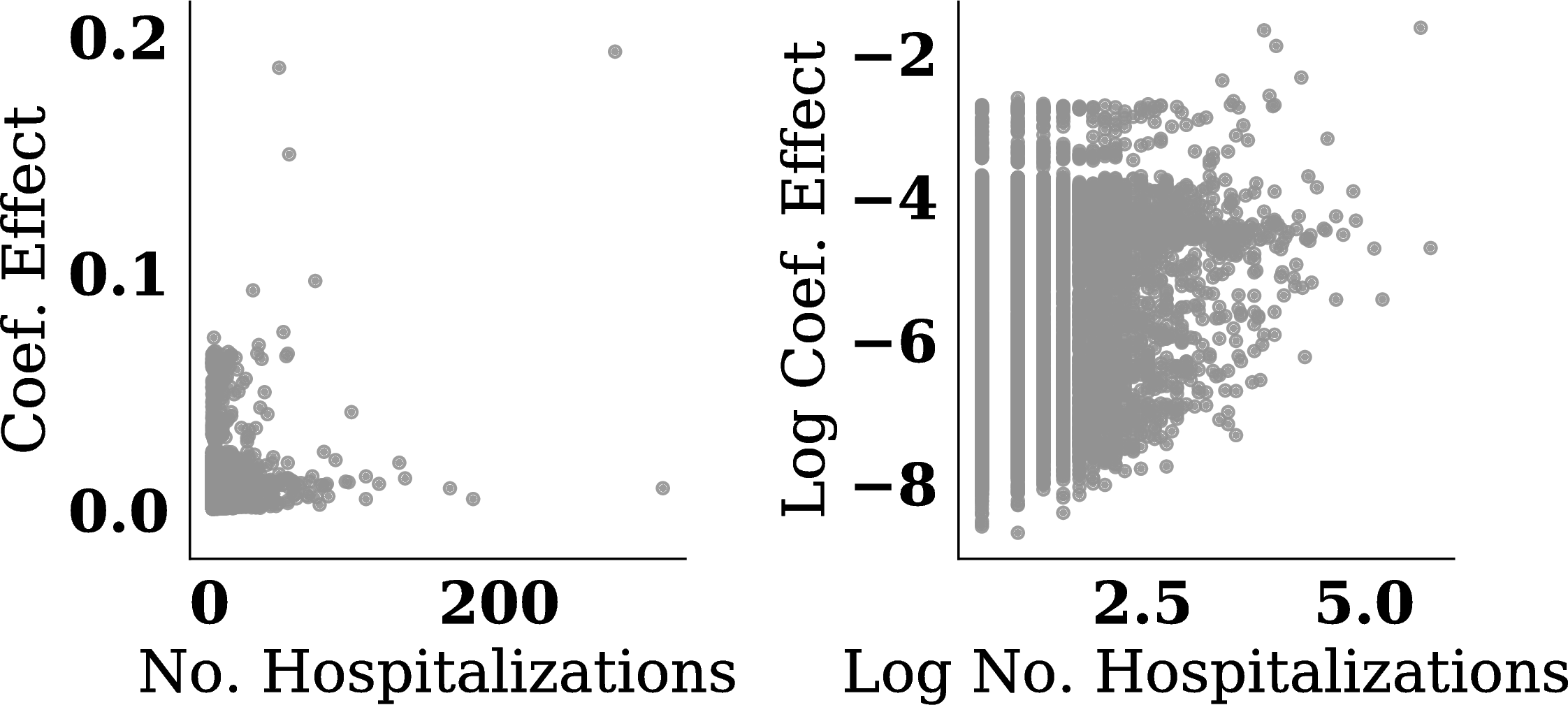

Supplement: S25 Fig — Influence over coefficients of HPLR1 vs. utilization is shown for each patient with two or more hospitalizations. Distance between coefficient vectors was computed using the l1 norm. (TIF) [file pone.0204920.s026.tif]
