# Supplementary material for: Predicting acute kidney injury at hospital re-entry using high-dimensional electronic health record data
Source: PLoS One. 2018 Nov 20;13(11):e0204920. doi: 10.1371/journal.pone.0204920 (PMC6245516; doi:10.1371/journal.pone.0204920)
Supplement: S1 Table — Shown is the choice of HP Alpha and the train, validation, and test mean squared error (MSE) of the regression from the fold in which Alpha was chosen. (PDF) [file pone.0204920.s027.pdf]

			Diagnosis	Race	Gender	Age
AKI + (N=5,618)	Alpha		0.015	0	0	0
	MSE	Train	0.021	0.32	0.032	0.030
		Validation	0.022	0.31	0.031	0.028
		Test	0.021	0.31	0.031	0.030
AKI - (N=84,395)	Alpha		1e-5	0	0	0
	MSE	Train	0.005	0.007	0.007	0.006
		Validation	0.005	0.007	0.007	0.006
		Test	0.005	0.008	0.008	0.006
