# Supplementary material for: Predicting acute kidney injury at hospital re-entry using high-dimensional electronic health record data
Source: PLoS One. 2018 Nov 20;13(11):e0204920. doi: 10.1371/journal.pone.0204920 (PMC6245516; doi:10.1371/journal.pone.0204920)
Supplement: S2 Table — For laboratory results, the first function is G, aggregation over hospitalizations, and the second is F, aggregation within a hospitalization; e.g., “mean max sCr” is the mean over hospitalizations of the maximum sCr of each hospitalization. (PDF) [file pone.0204920.s028.pdf]

RLR1 (+)	Mean (95% CI)
Age	0.3533 (0.3511, 0.3555)
Count Dx: AKI	0.083 (0.0816, 0.0845)
Count discharges with home organization care services	0.0774 (0.0761, 0.0787)
Mean max glucose	0.0741 (0.0722, 0.0761)
Mean mean urea nitrogen	0.0682 (0.0665, 0.07)
Mean max urea nitrogen	0.0612 (0.0597, 0.0627)
Count Dx: CKD	0.0448 (0.043, 0.0467)
Mean max potassium	0.0429 (0.0415, 0.0443)
Mean min direct bilirubin	0.0394 (0.0367, 0.0421)
Max mean urea nitrogen	0.0381 (0.0371, 0.0391)
Count Px: Assay of urine sodium	0.0369 (0.0352, 0.0387)
Mean max sCr	0.0325 (0.0299, 0.0352)
Max max urea nitrogen	0.0317 (0.0308, 0.0327)
Max mean sCr	0.0258 (0.0233, 0.0284)
Max max sCr	0.0253 (0.0237, 0.0268)
Min min direct bilirubin	0.0231 (0.0201, 0.0262)
Mean mean sCr	0.0218 (0.0186, 0.025)
Min mean urea nitrogen	0.0194 (0.0156, 0.0231)
Min max urea nitrogen	0.019 (0.0155, 0.0224)
Max max glucose	0.0177 (0.0155, 0.02)
**RLR1 (-)**	**Mean (95% CI)**
Mean min hemoglobin	-0.0855 (-0.0882, -0.0826)
Mean min glomerular filtration rate-caucasian	-0.0819 (-0.0846, -0.0793)
Max min hemoglobin	-0.0737 (-0.0785, -0.069)
Mean min albumin	-0.064 (-0.0653, -0.0627)
Mean mean albumin	-0.0617 (-0.0631, -0.0603)
Mean min chloride	-0.0579 (-0.0603, -0.0555)
Mean min calcium	-0.0502 (-0.0545, -0.046)
Min min glomerular filtration rate-caucasian	-0.0463 (-0.0477, -0.0449)
Marital status: single	-0.0341 (-0.0394, -0.0287)
Min min hemoglobin	-0.0239 (-0.0265, -0.0212)
Min min albumin	-0.0181 (-0.0204, -0.0156)
Min min chloride	-0.0177 (-0.0197, -0.0157)
Mean mean hemoglobin	-0.0096 (-0.012, -0.0071)
Sum count abnormally high sCr	-0.0045 (-0.0054, -0.0036)
Max mean albumin	-0.003 (-0.0045, -0.0013)
Max max potassium	-0.0027 (-0.0035, -0.0019)
Mean max albumin	-0.0022 (-0.0033, -0.001)
Max min calcium	-0.0021 (-0.0035, -0.0004)
Min mean albumin	-0.0019 (-0.0028, -0.0011)
Count “non-present” DRGs	-0.0015 (-0.0031, 0.0008)
