# Supplementary material for: Predicting acute kidney injury at hospital re-entry using high-dimensional electronic health record data
Source: PLoS One. 2018 Nov 20;13(11):e0204920. doi: 10.1371/journal.pone.0204920 (PMC6245516; doi:10.1371/journal.pone.0204920)
Supplement: S3 Table — For laboratory results, the first function is G, aggregation over hospitalizations, and the second is F, aggregation within a hospitalization; e.g., “mean max sCr” is the mean over hospitalizations of the maximum sCr of each hospitalization. (PDF) [file pone.0204920.s029.pdf]

RHPLR1 (+)	Mean (95% CI)
Age	0.3968 (0.3943, 0.4006)
Count Dx: AKI	0.132 (0.1302, 0.1339)
Max mean urea nitrogen	0.1181 (0.1153, 0.1208)
Max max urea nitrogen	0.0952 (0.0934, 0.0971)
Mean mean urea nitrogen	0.0837 (0.0797, 0.0879)
Max mean urea nitrogen	0.0754 (0.0737, 0.0771)
Mean max potassium	0.0565 (0.0501, 0.0628)
Sum count abnormally high sCr	0.0501 (0.0476, 0.0526)
Min mean urea nitrogen	0.0257 (0.0206, 0.0307)
Max max sCr	0.0198 (0.0151, 0.0243)
Count Dx: CKD	0.012 (0.0085, 0.0153)
Mean max sCr	0.0064 (0.0034, 0.0092)
Count Px: Assay of urine sodium	0.0052 (0.0027, 0.0075)
Sum count abnormally high glomerular filtration rate-caucasian	0.0047 (0.0036, 0.0059)
Mean max glucose	0.0033 (0.0005, 0.0055)
Count discharges with home organization care services	0.0027 (0.0004, 0.0045)
Count Px: Injection of furosemide or levetiracetam	0.0025 (0.0005, 0.0043)
Max max potassium	0.0012 (0.0, 0.0022)
Max count abnormally high sCr	0.0008 (-0.0001, 0.0014)
Sum count abnormally high glucose	0.0004 (-0.0004, 0.0007)
**RHPLR1 (-)**	**Mean (95% CI)**
Mean min hemoglobin	-0.1627 (-0.1675, -0.1577)
Mean min albumin	-0.1286 (-0.1322, -0.125)
Mean mean albumin	-0.0638 (-0.0695, -0.0582)
Max min hemoglobin	-0.0341 (-0.0404, -0.028)
Min min hemoglobin	-0.0307 (-0.036, -0.0254)
Mean min calcium	-0.005 (-0.0077, -0.0019)
Min min albumin	-0.0029 (-0.0046, -0.0009)
